# Pharmacological pain relief and women´s birth experience: a systematic review

**DOI:** 10.1186/s12884-025-07602-3

**Published:** 2025-04-26

**Authors:** Malin Ugarph Edfeldt, Hanne Gustavsson, Karin Hildén, Yang Cao, Helena Backman

**Affiliations:** 1 School of Medical Sciences, Örebro University, Örebro, SE S-701 82 Sweden; 2 Capio Health Center Ringen, Stockholm, SE S-118 60 Sweden; 3 Department of Obstetrics and Gynecology, Örebro University Hospital, Region Örebro County, Örebro, SE S-701 85 Sweden; 4 Clinical Epidemiology and Biostatistics, School of Medical Sciences, Orebro University, Orebro, SE S-701 82 Sweden; 5 Unit of Integrative Epidemiology, Institute of Environmental Medicine, Karolinska Institute, Stockholm, SE S-171 77 Sweden; 6 Department of Anaesthesia and Intensive Care, Region Örebro County, Orebro, S-701 85 Sweden

**Keywords:** Childbirth satisfaction, Birth experience, Pharmacological pain relief, Pregnancy, Labor

## Abstract

**Background:**

There is increasing interest in health care systems worldwide for maternal satisfaction with childbirth experience. The World Health Organisation (WHO) launched a recommendation 2018 regarding women’s right to equal and fair intrapartum care, where the importance of pharmacological pain relief was highlighted. Our objective with this systematic review was to summarize and assess the current knowledge regarding the impact of obstetric pharmacological pain relief on maternal satisfaction with childbirth.

**Methods:**

The databases Pub Med, Cochrane, EMBASE and CINAHL were searched for studies in the English language published after 1998 that investigated the effect of pharmacological pain relief on women´s birth experience after vaginal delivery. Studies reporting assessments of subjective satisfaction with childbirth in women planned for vaginal delivery were selected. The results were summarized narratively. For studies where comparable association measures were available, forest plots are presented. Due to heterogeneity of research questions and indirectness of measuring instruments, no meta-analyses were performed.

**Results:**

A total of 15,136 women were included from 18 studies. Two randomized controlled studies, nine cohort studies, six cross-sectional studies and one case control study, all had a moderate or high risk of bias. The studies used inconsistent methods to measure outcomes; therefore, no conclusion could be drawn regarding a possible correlation between pharmacological pain relief and overall birth experience.

**Conclusions:**

This systematic review could not show a correlation between pharmacological pain relief and women´s experiences of childbirth, mainly due to large heterogeneity between studies. To evaluate pain relief during labour and improve women´s childbirth experiences, high-quality research is warranted.

**Trial registration:**

The study was registered in PROSPERO (prospective register of systematic reviews) 18 Dec 2018 (ID 116744).

**Supplementary Information:**

The online version contains supplementary material available at 10.1186/s12884-025-07602-3.

## Background

Maternal satisfaction with birth experience is of great importance for both the individual and society [1]. A negative experience affects the early connection between mother and child and can further lead to Post-Traumatic Stress Disorder (PTSD) and fear of birth in future pregnancies [2, 3], with an increasing number of requests for operative deliveries [4–6]. Health care systems worldwide, including care during pregnancy, have moved from authority-based organizations towards an increasingly patient-focused approach in recent decades [7–9].

A large diversity of aims, definitions and outcomes in studies of birth satisfaction reflect the complexity of childbirth experience [10–15]. Some authors promote nonmedical births [16, 17], and some have a positive attitude towards pharmacological treatment, focusing on the effectiveness of reducing actual pain, but not looking at the overall satisfaction with birth experience [18–22]. A secondary outcome in studies with a nonmedical focus is often in fact a decreased need for pharmacological pain relief [23–26], introducing a bias that makes it difficult to evaluate a possible positive effect from medical pain treatment. A previously published systematic review excluded observational and qualitative studies, which we believe may be misleading, as the variables of pain relief and birth experience are not always most appropriately studied using a randomised approach. The World Health Organization (WHO) makes an important statement in the WHO Recommendations “Intrapartum care for a positive childbirth experience” from 2018 [1], concluding that labouring women have the right to pain relief when giving birth. The rate of epidural analgesia usage in the obstetric ward continues to increase in high income countries [27]. In 2014, 37% of women giving birth in Sweden received an epidural, compared to 48% in 2024 [28]. We believe such a trend of increased use of anaesthetic resources needs to be further evaluated.

Participation in decision-making during labour and close contact with the midwife have been demonstrated to be crucial elements for a positive birth experience [29–32]. Other significant elements for a positive birth experience include timing of assessment [33–35], with women generally rating their experience most positively immediately postpartum, which can be explained by a ‘halo effect’ due to having a healthy child [33]. Additionally, the parity of the woman plays a role, as nulliparous and multiparous women tend to respond to different aspects of childbirth [36]. None of the factors of pain relief [13, 37, 38], pain itself [39], mode of delivery [10, 40], medicalized level of care [11, 40, 41], or obstetric interventions [42, 43] offers a simple solution to improve birth experience, as the results are contradictory [30, 35, 43, 44]. It is not clear whether the negative birth experience reported when given pain relief depends on the need for pain relief [34, 35], less support from midwives after pain relief [13, 45], or if it is due to the analgesic method itself.

Through this systematic review, we aim to summarise research conducted in this area and investigate whether an association can be identified between pharmacological pain relief and maternal birth experience.

## Methods

The systematic review was conducted according to the Preferred reporting Items for Systematic Reviews and Meta-Analyses (PRISMA) guidelines [46, 47], recommendations by the Swedish Agency for Health Technology Assessment and Assessment of Social Services (SBU) [48], the Cochrane Collaboration tools [49] and the Grading of Recommendations, Assessment, Development and Evaluations (GRADE) guidelines [50, 51]. The study was registered in Prospective Register of Systematic Reviews (PROSPERO) 18 Dec 2018 (ID 116744). The literature search was carried out with the assistance of an information specialist starting on 11 Feb 2019, with the last update conducted in January 2023. Three search strings were applied: (1) Labor, Delivery, Parturition. (2) Pain, Analgesia. (3) Patient satisfaction. The complete search strategy (Additional file 1) and the PRISMA checklist (Additional file 2) are available as supplementary information.

### Search strategy

**Fig. 1:**
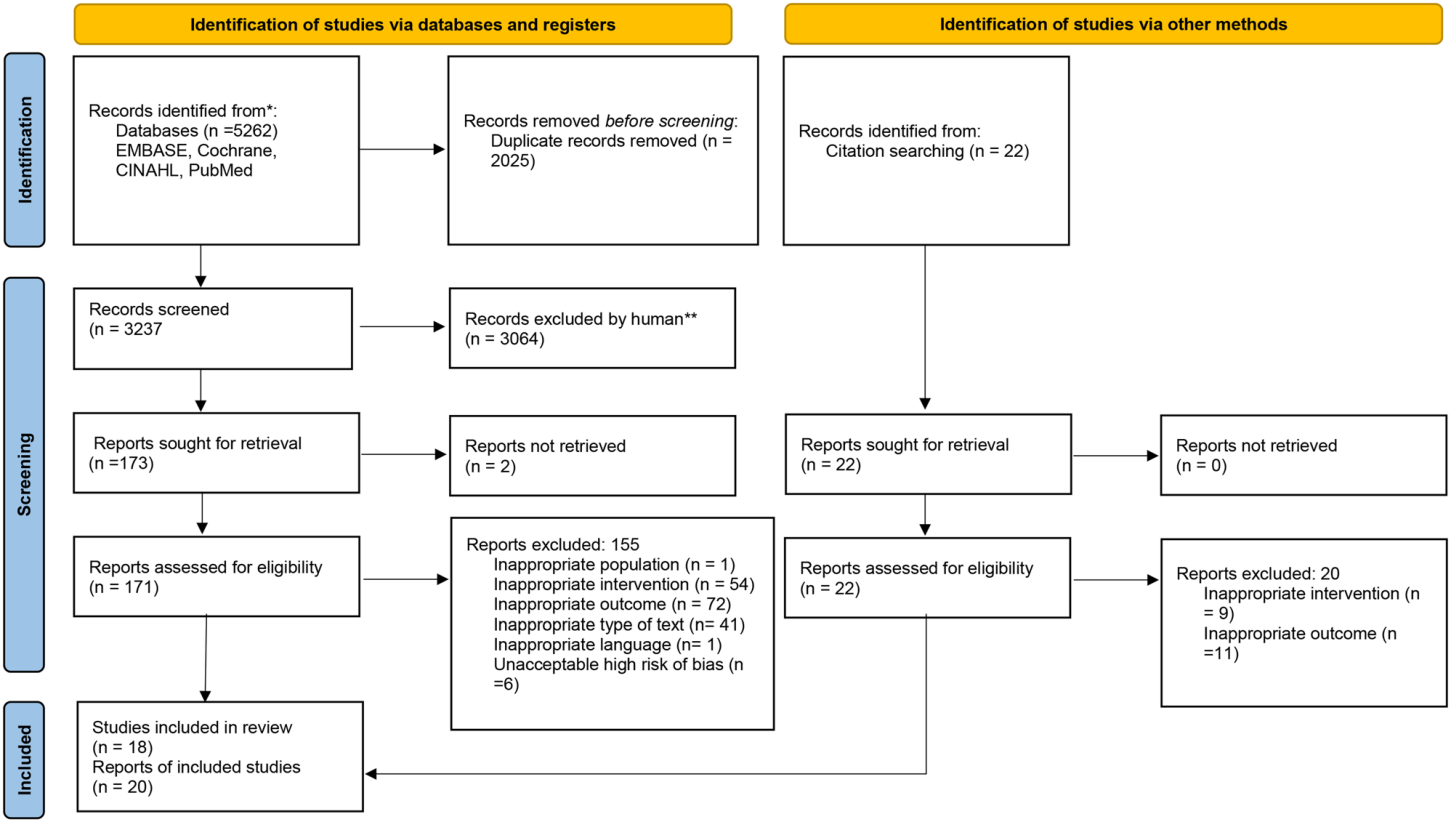
PRISMA Flow chart of study selection. Three studies contained multiple intervention groups, leading to 21 total comparisons in the analysis

An electronic systematic literature search was performed in the four databases PubMed, CINAHL, EMBASE, and the Cochrane Library. Limits for language (English) and date from 1998-01-01 were applied for the search. No limits were set due to study design. The first searches were made before the PRISMA update presented in 2021 [46, 47], why we present the results in total (Fig. 1), and not number of papers for each database. Reference lists of retrieved articles and systematic reviews that were found through the search process were screened for additional titles. Editorials, commentaries, study protocols, pilot studies and conference abstracts were excluded. Information that was not supplied in the original articles has not been requested from the authors.

All articles identified were independently screened by two reviewers (MUE and HG) using the reference management program Rayyan QCRI (http://rayyan.qcri.org) [52]. Articles were first screened for title and abstract, according to a study protocol with defined inclusion and exclusion criteria. If the reviewers were of different opinions during the selection phase, the paper in question was assessed in full text. Selected articles were retrieved in full text and assessed for eligibility. Any disagreements after assessing papers in full text were resolved by discussion with a third author (H.B.). A PRISMA flow chart is presented in Fig. 1.

### Selection criteria

The study population comprised women who had given birth by vaginal delivery in a hospital. Studies were included where pharmacological pain relief had been administered to women during vaginal delivery. Comparator groups consisted of women not receiving pharmacological pain relief. We defined pharmacological pain relief as administration of opioids, local anaesthetics given neuraxially or as infiltration, non-steroidal analgesics and inhaled nitrous oxide. The outcome in eligible studies was women´s subjective evaluation of their birth experience. Studies where outcome was satisfaction with care and studies evaluating pain relief, not overall satisfaction with birth experience, were excluded.

### Data extraction

Data were extracted for general information (author, year of publication, country, study design, sample size, parity and age of participants), information of comparator group without pharmacological pain relief, method used to evaluate satisfaction with birth experience and timing of assessment of birth experience. We considered it to be an early assessment if less than two months had passed since delivery, and a later assessment if two months or more had passed, based on knowledge of how women´s description of satisfaction with birth experience changes over time [53–55]. Studies that did not meet the eligibility criteria or those that could not be found in full text, were excluded with reported reasons (Additional file 3).

Evaluation of birth experience had different labels in the included studies and will be used interchangeably in this text, e.g., maternal satisfaction, birth experience, recall of birth and satisfaction with childbirth.

**Fig. 2:**
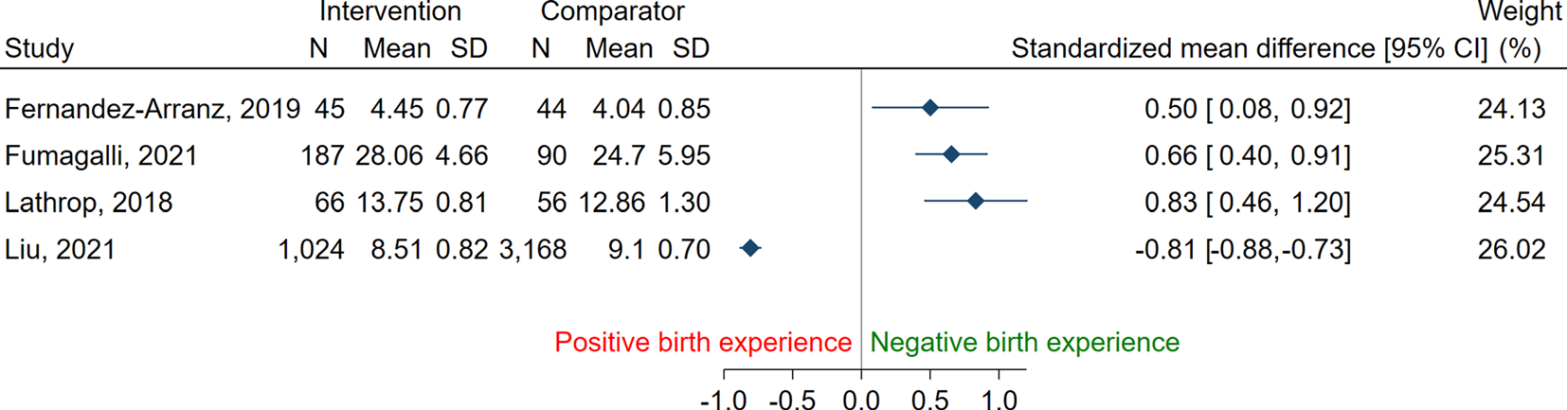
Birth Experience in women receiving pharmacological pain relief (intervention) versus women without (comparator) Data from studies using continuous variables, presented as Standardised Mean Difference. No pooling of results due to large heterogeneity between studies

**Fig. 3:**
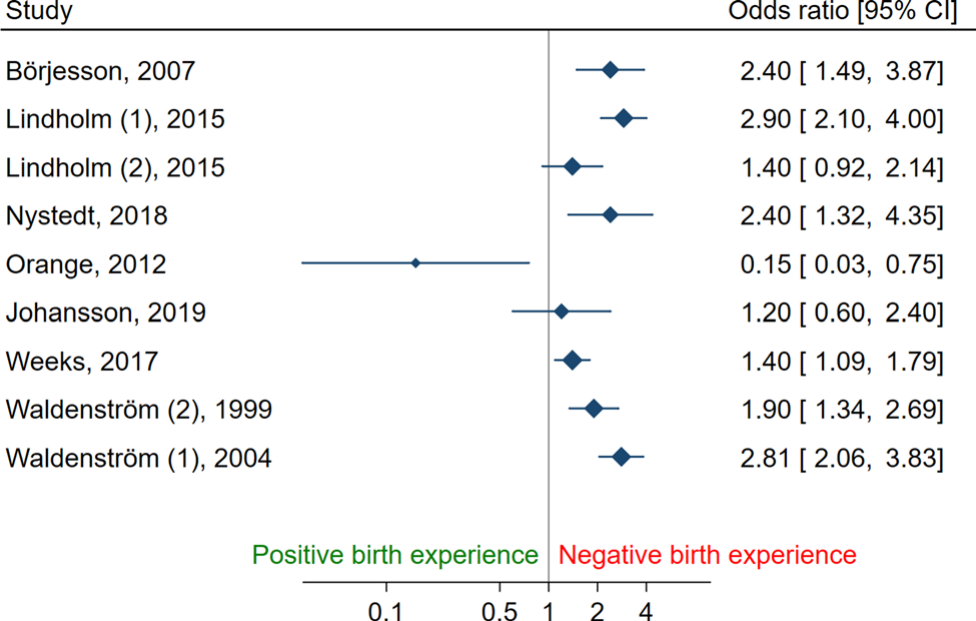
Birth Experience in women receiving pharmacological pain relief (intervention) versus women without (comparator) Data from studies using categorical variables, presented as Odds Ratio. No pooling of results due to large heterogeneity between studies. *NOTE: Results presented on log scale*

Results are presented using forest plots (Figs. 2 and 3) for studies where data were available for the calculation of relevant association measures. No meta-analyses were performed due to significant heterogeneity and moderate to high risk of bias in the studies. Where numerical data were not available for calculation for a forest plot, the results are presented narratively only. The statistical software STATA 17.0 was used for data visualization.

All but two studies [56, 57] presented “a positive birth experience” as reference value for the dependent variable. To be able to compare with the other trials, we calculated an inverse odds ratio for these two studies.

### Quality and risk of bias assessment

**Fig. 4:**
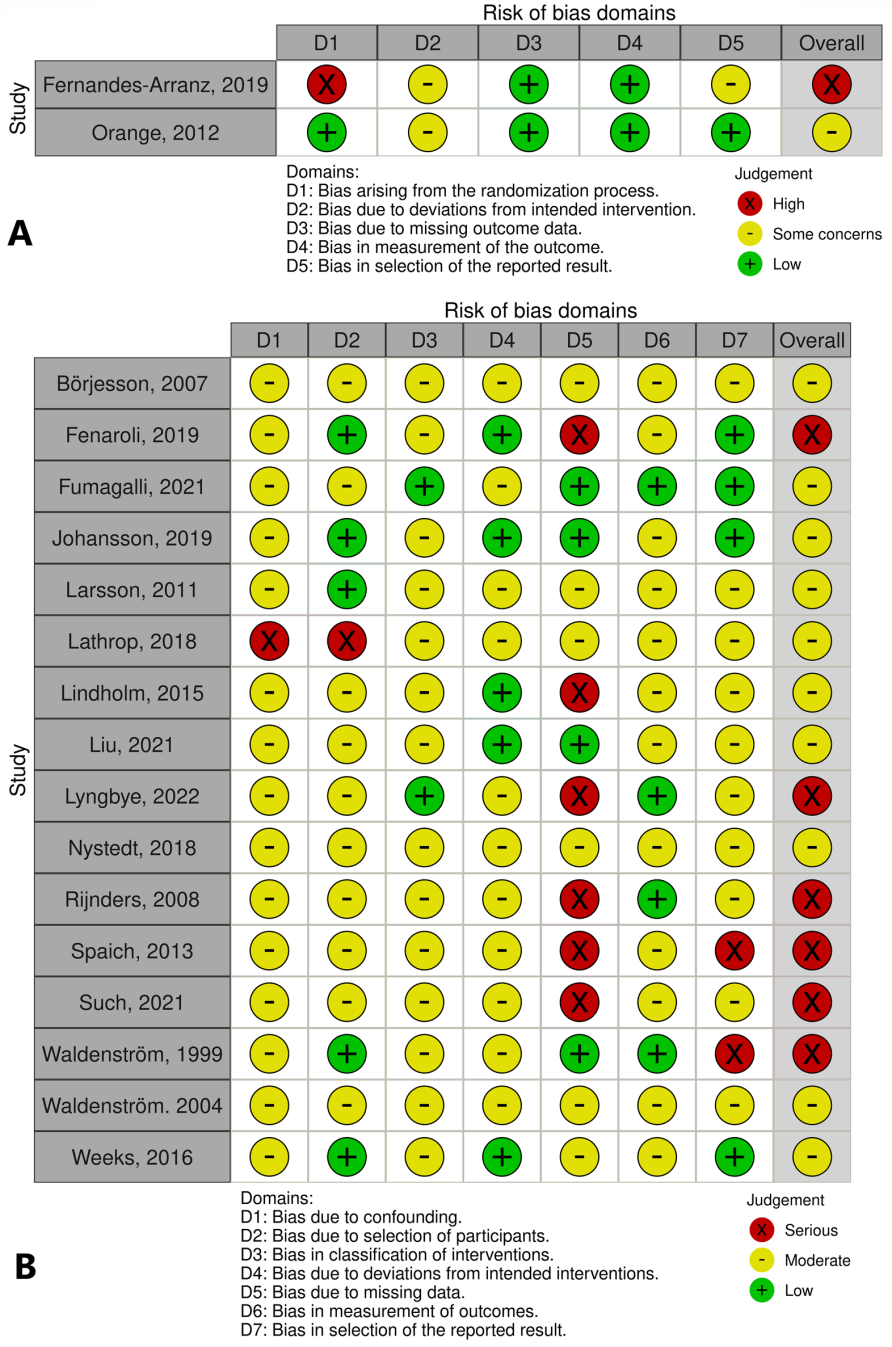
Risk of bias. **A**: Included RCT´s. **B**: Included observational studies. Review authors’ judgements about each risk of bias item for each included study

The eligible studies were controlled for risk of bias independently by two researchers (MUE, HG) based on recommendations and protocols from the Swedish Agency for Health Technology Assessment and Assessment of Social Services (SBU) [48]. The SBU protocols are Swedish translations of the Cochrane Collaboration tools [49]. Risk of Bias 2 (RoB 2) [58] were applied for included randomized trials and Risk of Bias In Non-Randomized Studies (Robins-I) [59] was used for non-randomized trials. We assessed the included randomized controlled trials (RCTs) for risk of selection bias, performance bias, attrition bias and reporting bias. Eligible non-randomized studies were assessed for risk of confounding, biased selection of participants, biased classification of interventions, deviation from intended interventions, missing data, biased measurements of outcome and biased selection of reported results. Trials where no stratification or regression analysis where made to control for confounding factors were excluded with the reason ‘unacceptable high risk of bias’. A summary of risk of bias assessment is presented in Fig. 4a and b using the Risk of Bias VISualization tool (Robvis) [60]. To assess publication bias, funnel plots were constructed (Additional files 6 and 7). For an overall assessment of quality and relevance, the GRADE protocol [50, 51] was applied. This model uses five considerations when assessing evidence: study limitations, consistency of effect, imprecision, indirectness and publication bias. (Additional file 4)

## Results

### Systematic search

A PRISMA flowchart describes the selection process (Fig. 1). The search of electronic databases resulted in 3206 records after duplicate sorting. Following the screening of titles and abstracts, 173 articles were retrieved from databases, supplemented by an additional 22 records obtained through manual search of reference lists, resulting in a total of 195 articles collected in full text. Two reports could not be found in full text. The reasons for exclusion at abstract level were inappropriate population (caesarean section or non-obstetric surgical interventions), inappropriate intervention (no pharmacological pain relief wasused) or inappropriate outcome (only pain score was evaluated, no measure of overall birth experience). Reasons for exclusion after assessment in full text are supplied in Additional file 3. Eighteen studies were found to be eligible and included in the systematic review, where of three studies presented two intervention groups [61–63]. Each intervention group were analysed as a separate comparison in the synthesis, giving in total 21 analyses.

### Study characteristics

Description of included studies are presented in Table 1. Of the eligible studies, there were two RCTs [56, 64], eight prospective cohort studies [13, 37, 44, 57, 61, 62, 65, 66], one retrospective cohort study [67], six cross-sectional studies [63, 68–72] and one case‒control study [73]. No qualitative study fulfilled the inclusion criteria.

**Table 1:** Description of included studies

Author Year	Country	Sample size Study design	Study population	Intervention (% of total) and comparator	Birth Experience ¤	Assessment tool ^#^/ time of assessment post-partum	Comments
***Higher maternal satisfaction with pharmacological pain relief***							
Orange [56] 2012	Brazil	70 Randomized controlled study	P*and NP** Term pregnancies 100% vaginal births. Mean age 22 years.	Combined spinal epidural analgesia (CSE) (50%) compared to non-pharmacological pain relief	Higher maternal satisfaction among women receiving combined spinal epidural analgesia (CSE)	1–5 Likert scale / immediately	No statistically significant difference between groups in rate of CS^##^, instrumental birth, oxytocin induction, duration of expulsion phase or neonatal outcomes
Liu [70] 2021	China	4192 Cross sectional study	P* and NP** Gestational age > 32 weeks. 100% vaginal births Mean age 29,8 years	Epidural analgesia (EDA^§^) (75,6%) compared to no EDA^§^	Higher maternal satisfaction among women receiving epidural analgesia (EDA^§^)	1–10 Likert scale /2–3 h	Duola or family member present, use of breathing technique and warm perineal compress were other factors associated with higher maternal satisfaction
***Lower maternal satisfaction with pharmacological pain relief***							
Börjesson [66] 2007	Sweden	518 Prospective co-hort study	NP** 89% Vaginal births. Mean age 27,2 years	EDA^§^ (65%) compared to no EDA^§^	Higher odds for a negative birth experience among women receiving EDA^§^	VAS 1–100 mm /within 3 months	Elective CS^##^ were excluded. Other modes of delivery than unassisted vaginal birth were associated with a higher risk of a negative birth experience
Fenaroli [65] 2019	Italy	111 Prospective longitudinal study	NP** Term pregnancies. 80.6% vaginal births. Mean age 32 years	EDA^§^ (45,4%) compared to no EDA^§^	Less positive birth experience among women receiving EDA^§^	W-DEQ (B) ^#^ / 1–5 days	Elective CS^##^ were excluded No significant association between birth experience and mode of delivery or induction of labor
Fernández-Arranz [64] 2019	Spain	89 Randomized controlled study	P* and NP** 100% Vaginal births. Mean age 32,9 years	Haloperidol and Pethidine (49%) compared to use of birthing ball	Lower maternal satisfaction among women who received Haloperidol and Pethidine	Mackey Satisfaction Childbirth Rating Scale ^#^ /before discharge	No blinding of participants or investigators
Fumagalli [71] 2021	Italy	277 Cross sectional study	P* and NP**. Term pregnancies. 94,4% vaginal births. Mean age 33 years	EDA^§^ (32%) compared to no EDA^§^	Lower maternal satisfaction among women who received EDA^§^	BSS-R ^#^ /before discharge	Active phase of labor > 12 h and vacuum assisted birth was associated with a lower birth satisfaction
Johansson [68] 2019	Sweden	584 Cross sectional study	NP** 86.5% vaginal births. Mean age 29 years	EDA^§^ (35%) compared to no EDA^§^	Higher odds for a negative birth experience among women receiving EDA^§^	1–10 Likert scale / immediately	Oxytocin induction significantly associated with use of EDA^§^ Mixed groups: Morphine, nitrous oxide and EDA^§^ not separated
Lathrop [73] 2018	USA	122 Case control study	P* and NP** Term pregnancies 100% vaginal births Age > 18 years	EDA^§^ (46%) compared to water birth	Less positive childbirth experience among women receiving EDA^§^	CEQ ^#^ / 1–7 days	Primary aim of study was to explore benefits with water birth compared to conventional birth
Lindholm [61] 2015	Sweden	866 Prospective longitudinal study	P* and NP** 82% vaginal births. Majority aged 25–35 years	EDA^§^ (32%) compared to no EDA^§^ Nitrous oxide (85%) compared to no nitrous oxide	Less positive birth experience among women receiving EDA^§^ No association found between nitrous oxide and birth experience	1–5 Likert scale / 2 months	Elective CS^##^ were excluded. Primary aim of study was to identify factors associated with preferred pain relief methods
Lyngbye [62] 2022	Denmark	201 Prospective cohort-study	P* and NP** Term pregnancies 92% vaginal births Mean age 29,2 years	EDA^§^ (26,7%) compared to no EDA^§^ Nitrous oxide (47%) compared to no nitrous oxide	Use of EDA^§^ was associated with a led positive childbirth experience Use of nitrous oxide was associated with a less positive childbirth experience	CEQ ^#^ /6 weeks	52% of the women reported a lower satisfaction score after 6 weeks compared to after 1 week
Nystedt [74] 2018	Sweden	928 Cross sectional study	P* and NP** Uncomplicated pregnancies 82% vaginal deliveries Majority aged 25–35 years	Obstetric analgesia (31%) compared to no obstetric analgesia	Higher odds for a negative birth experience among women receiving obstetric analgesia	Likert scale 1–5 /2 months	No definition of the term “obstetric analgesia” Other modes of delivery than unassisted vaginal birth, were associated with a higher risk of a negative birth experience
Waldenström [57] 1999	Sweden	1111 Prospective longitudinal study	P* and NP** 84.3% vaginal deliveries. Mean age 30 years	Nitrous oxide (33%) compared to no nitrous oxide	Use of nitrous oxide was associated with a a negative birth	Likert scale 1–7 / 2 months	No association found for EDA^§^ or Pethidine Mixed groups: EDA^§^, Pethidine and Nitrous oxide not separated
Waldenström [13] 2004	Sweden	2541 Prospective longitudinal study	P* and NP** 92% vaginal births. Mean age 29.5 years	EDA^§^ (30%) compared to no EDA^§^	Higher risk for a negative birth experience among women receiving EDA^§^.	Likert scale 1–7^#^ / 2 months	Emergency CS^##^, instrumental birth, induction, strong pain, not part of decision-making and child to neonatal unit were correlated to a risk of a negative birth experience
Weeks [69] 2017	Chile	1534 Cross sectional study	P* and NP**. 80% vaginal births Age 18–32 years	Pharmacological pain management (74%, nitrous oxide or EDA^§^) compared to no pharmacological pain management	Lower maternal satisfaction among women receiving pharmacological pain management	Maternal well-being scale, by Uribe et al. 42-questions ^#^ / 1–2 days	CS^##^, continuous monitoring of fetal heartbeat and episiotomy were associated with a lower maternal satisfaction
***No association found between maternal satisfaction and pharmacological pain relief***							
Larsson [37] 2011	Sweden	280 Prospective longitudinal study	NP** at term. 56% vaginal births Age not supplied	EDA^§^ (26,3%) compared to no EDA^§^	No association found between EDA^§^ and birth experience	W-DEQ (B)^#^ / 9 months	Secondary aim of study was to compare the assessment tools W-DEQ (B) and VAS Mixed population, evaluating both vaginal births and caesarean section
Rijnders [67] 2008	Netherlands	1293 Retrospective cohort study	P* and NP** 86.9% vaginal births Mean age 31 years	Pain relief (14%) compared to no pain relief	No association found between pain relief and recall of birth experience	One question from “Greater Expectations” ^#^ / 3 years	Assisted vaginal, non-elective CS^##^, referral during birth, no choice of pain relief and dissatisfaction with coping with pain were associated with a negative recall of child-birth No information supplied regarding type of pain relief
Spaich [44] 2013	Germany	335 Prospective longitudinal study	P* and NP**. At term pregnancies. 65% vaginal birth. Median age 31 years	Intravenous pain medication and EDA^§^ compared to no pain relief	No association found between pharmacological pain relief and birth experience	Salmon Item List^#^ /Before discharge from hospital	High level of pain perception was associated with risk for a negative experience No significant association found between mode of delivery and birth experience
Such [63] 2021	USA	84 Cross sectional study	P* and NP**. At term Uncomplicated pregnancies. 100% vaginal births. Age > 18 years.	EDA^§^ (33%) compared to no epidural analgesia Nitrous oxide (33%) compared to no nitrous oxide	No significant difference in satisfaction with birth experience between groups No significant difference in satisfaction with birth experience between groups	BSS-R^#^ /within 6 h	Income and education were independently associated with a higher birth satisfaction score

* P = Parous

**NP = nulliparous

^§^ EDA = epidural analgesia

^¤^ Birth Experience was labelled by authors as follows: Maternal satisfaction, Childbirth experience, Satisfaction with birth experience and Recall of birth experience

^#^ Assessment tool measuring Birth Satisfaction or Birth Experience. For more information see Additional file 5 (VAS, WDEQ-B, Mackey Satisfaction Childbirth Rating Scale, Greater Expectations, BSS-R, CEQ, Salmon Item List)

^##^ CS = Caesarean section

Altogether, 15,136 women aged between 16 and 47 years participated in the studies reported in this systematic review. Seven of the included studies were conducted in Sweden (6828 women, 45%) [13, 37, 57, 61, 66, 68, 72], one in China (4192 women, 28%) [70], one in Chile (1534 women, 10%) [69], one in the Netherlands (1293 women, 8,5%) [67], two in Italy (388 women, 2.6%) [65, 71], one in Germany (335 women, 2,2%) [44], two in the USA (206 women, 1,4%) [63, 73], one in Denmark (201 women, 1,3%) [62], one in Spain (89 women, 0,5%) [64] and one in Brazil (70 women, 0,5%) [56].

Foetal or maternal medical concerns were exclusion criteria in nine studies [44, 56, 63, 64, 68, 69, 71–73] and 14 studies excluded women due to language barriers [13, 37, 57, 61–65, 68, 70–74]. In 13 studies [13, 37, 44, 57, 61, 62, 65–69, 71, 72], 3,6–44% of women were delivered with caesarean section, where the majority were non-elective. Rijnders et al. [67] included 42% homebirths. All other studies were hospital-based only.

Sixteen of the included studies received ethical approval of the study protocols. According to Rijnders et al. [67], no ethical approval is needed in the Netherlands if no invasive procedures are involved. No information on ethical approval was supplied by Waldenström et al. 1999 [57].

The interventions of interest for this systematic review were pharmacological pain relief. For six of the included studies, assessing the effect of pharmacological pain relief on birth experience was a secondary aim. The primary aim of these articles were to assess birth experience related to water birth [73], mode of delivery [44] or medical interventions in general during labour [13, 37, 57, 65].

### Assessment of birth experience

Numerical data were not available to present graphically in six of the included studies [37, 44, 62, 63, 65, 67] and are reported narratively. Results from 12 studies, with 13 groups, are presented in forest plots (Figs. 2 and 3).

Women receiving pharmacological pain relief were more likely to have a negative birth experience in 12 of the included studies [13, 57, 61, 62, 64–66, 68, 69, 71–73], a more positive experience in two studies [56, 70], while no association was found in four studies [37, 44, 63, 67] after adjusting for confounders (Table 1). Of the articles where pharmacological pain relief was reported to be a predictor for a negative birth experience, eight studies included women with labour epidural analgesia [13, 61, 62, 65, 66, 68, 71, 73], two studies used unspecified pharmacological pain management [69, 72], one study used Haloperidol and Pethidine [64], and three studies offered Nitrous Oxide [13, 61, 63]. Combined Spinal Epidural (CSE) analgesia in one randomized controlled study [56] and epidural analgesia in one cross-sectional study [70] were correlated with higher maternal satisfaction. The majority of women in all of the included studies, regardless of receiving pharmacological pain relief or not, reported a positive birth experience.

Timing of assessment of birth experience in the included studies varied from one hour to three years postpartum. When comparing studies that assessed birth experience within two months after childbirth [44, 56, 62–65, 68–71, 73] versus studies assessing after two months or more [13, 37, 57, 61, 66, 67, 72], no clear difference associated with timing of evaluation could be shown. However, the two articles showing the most positive effect of pain relief on birth experience [56, 70] used an early assessment. As methods of assessment of outcome varied between the studies, no overall statistical comparison could be made.

Different instruments to measure overall experience with childbirth were used in the included studies. A summary of assessment tools found in this systematic review is presented in Additional file 5. Furthermore, the definition of a negative experience varied, with some authors categorising women reporting a neutral experience as having a negative experience, while other authors categorised this group as having a positive experience. It should be acknowledged that more than 30 other instruments to measure satisfaction with birth experience are available [75–78].

### Quality and bias assessment

Of the 171 studies found through search in databases and assessed for eligibility, six studies were excluded due to unacceptably high risk of bias, as no regression analysis was performed to control for relevant confounders. (Fig. 1, Additional file 3)

**Fig. 5:**
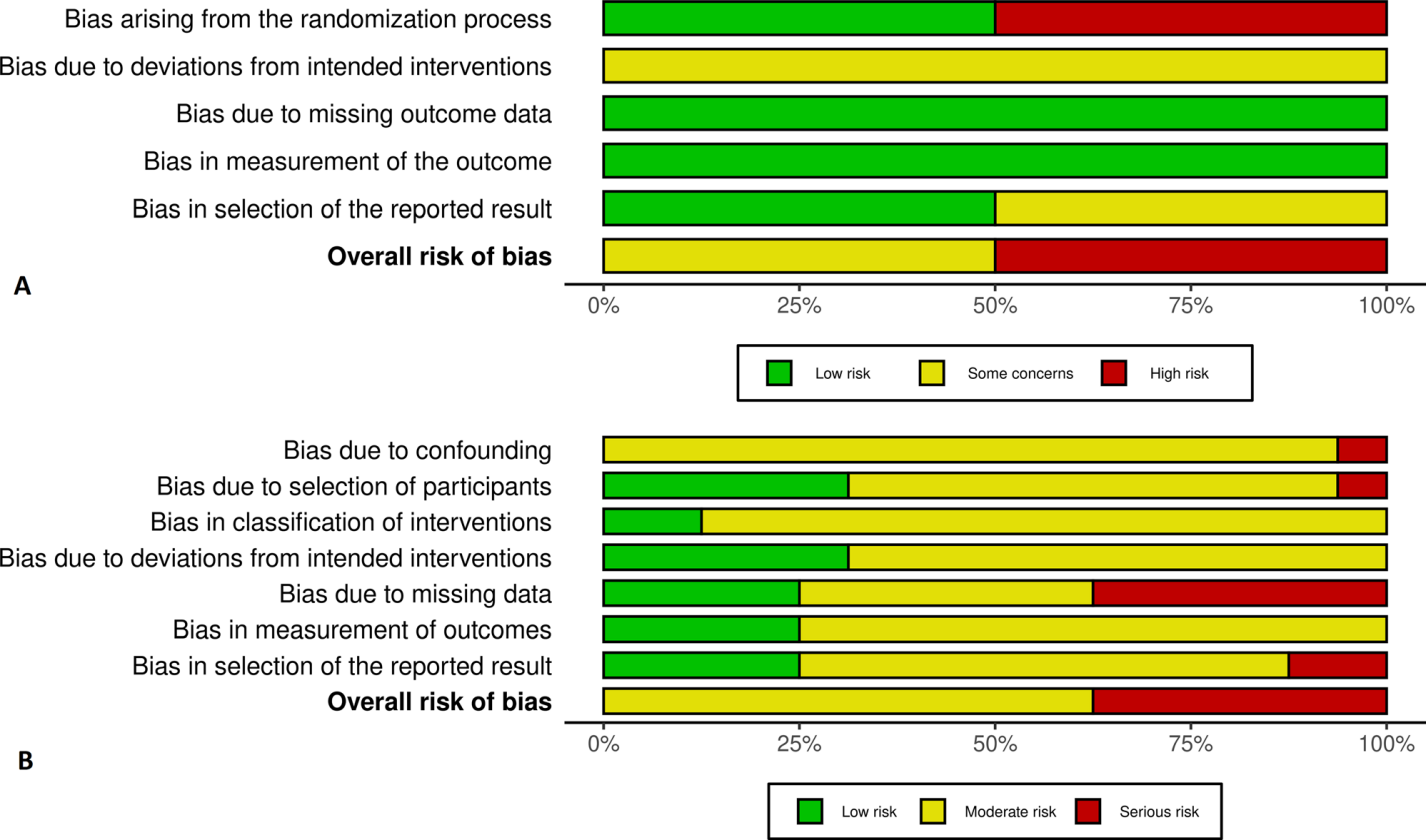
Risk of bias. Summary plot. **A**: Included RCTs. **B**: Included observational studies. Review authors’ judgements about each risk of bias item presented as percentages across all included studies

Nine of the included studies were assessed as having an overall high risk of bias, mainly within the domain ´missing data´, due to low response rate of approached participants (> 25%) [44, 61–63, 65, 67] and due to lack of control for possible confounding factors [73]. Moderate risk of bias was assessed in nine studies, where five studies had issues with selection bias due to missing information of background characteristics [37, 57, 65, 68, 69]. No study was assessed as having a low risk of bias (Figs. 4 and 5).

Egger test for categorical data showed beta = -0.53, z = -0.22, p-value = 0.822, indicating no statistically significant evidence of small-study effects. For continuous data, Egger’s test did not indicate statistical significance, beta = 3.00, z = 0.94, p-value = 0.349, however, due to less than ten studies, we refer to a funnel plot. The funnel plot showed obvious asymmetry, suggesting potential publication bias. The asymmetry may also be due to poor methodological design in the included studies and the language restriction applied during the literature search. For categorical data the funnel plot generally appeared symmetric and Egger’s test was not statistically significant, indicating no substantial publication bias. (Additional file 6 and 7).

Test of homogeneity for continuous results gave I^2^ = 94.78%, Q = 217.74, p-value < 0.001, and for categorical results I^2^ = 99.49%, Q = 464.54, p-value < 0.001, indicating statistically significant high heterogeneity regarding the primary outcome, women´s birth experience.

Furthermore, GRADE assessment showed an inconsistency in study design, study population, timing of evaluation and methods to measure birth experience between the included studies resulted in downgrading of evidence (Additional file 4).

No conclusion could be drawn regarding an association between birth experience and pharmacological pain relief after quality and bias assessment.

## Discussion

In this systematic review we report an association between a negative birth experience and received pharmacological pain relief in 12 of the included studies (*n* = 8882), compared to approximately half the number of women (*n* = 4262) who reported a positive experience after given pain relief, and no association was found for 1992 women. Despite these numbers, we could not draw a conclusion regarding an association between pharmacological pain relief and birth experience, due to the considerable heterogeneity among the studies, as well as a substantial variation in methods to measure birth experience. Eight different instruments were found among the included studies, where dichotomized scales varied significantly regarding definition of a negative birth experience.

The overall quality of evidence was downgraded due to heterogeneity of definitions of birth experience and assessment tools, with the result that meta analyses were not possible to perform in an adequate manner.

### Interpretation

The mixture of objectives and aims among the included studies highlight the fact that childbirth experience involves more aspects of labour and healthcare, than only pain relief. Unbearable pain has been shown to be an independent risk factor for negative birth experiences [29, 72, 79, 80]. It is plausible that women experiencing severe pain are more frequently provided with pain relief, why women’s ratings of birth experience after having received pain relief, instead may reflect their perceptions at the peak of pain. This theory is supported by one of the articles included in this systematic review, in the multivariate analysis by Waldenström et al. [13], who showed how pain, and not epidural analgesia, negatively affected satisfaction. The authors discuss whether pain relief might act as an intermediate variable for pain itself in the women´s recollection of their childbirth experience. In addition, women with strong anxiety tend to use more pharmacological pain relief, and regardless of receiving analgesia, they have more negative birth experiences [61, 81]. One of the RCTs in our review showed that combined spinal epidural (CSE) had a positive effect on birth satisfaction [56], even though the two groups reported the same baseline pain. This result reconfirms how pain can be a confounder in observational studies.

Liu´s study from China [70], which included 4192 women, reported a highly significant positive effect of epidural analgesia on maternal satisfaction, compared to the other similar studies. There are several possible interpretations for this outlying outcome. The Chinese study was conducted in a specialized maternity centre with focus on continuity of care, a recognized factor associated with a positive birth experience. Liu´s study is 15 to 50 times larger than other included studies in the review, which strengthens the reliability of the study´s result.

In contrast to our study, Ghanbari-Homaie et al. [82] found in their systematic review a positive effect of pharmacological pain relief on birth experience. There are some possible explanations for this difference in outcome. We included all study designs in our study, whereas Ghanbari-Homaie et al. included only RCTs. Clearly, there are ethical dilemmas in research involving childbirth. A double-blinded randomisation comparing vaginal delivery with pain relief to childbirth without pain relief is not a feasible option, which is why observational study designs predominate in this area. A double-blinded randomization comparing vaginal delivery with pain relief to childbirth without pain relief is not an ethically feasible option, which is why observational study designs predominate in this area. Ghanbari-Homaie et al. included both studies looking at satisfaction with the pain relief, as well as overall birth experience. Furthermore, their study included articles comparing different forms of pharmacological pain relief. In our review, the comparator groups did not receive any pharmacological pain relief.

We did not find an obvious difference in birth satisfaction between studies assessing the experience within two months [44, 56, 65, 68, 73] compared to after two months to three years after childbirth [13, 37, 57, 61, 67]. This is not fully in accordance with previously published results, where the timing of assessing birth experience has been shown to be an influential factor [83, 84] The discrepancy may be attributed to the small number of included articles and the heterogeneity of the assessment tools used in the included studies. Some authors looking at the effect of timing of assessment refer to the halo effect, an initial overwhelming feeling of gratefulness for the baby and possibly a denial of the recent pain. This would explain the higher percentage of values correlating to a “positive experience” directly after giving birth. According to some authors, most attention should be given to ratings lower than “very positive experience” in early evaluation [33, 84, 85], as these could indicate a more negative recall of the experience later.

Measuring childbirth experience is complex, as evidenced by the large number of instruments and scales available [75, 76, 78]. Most instruments aim to identify aspects of childbirth possible to control in this overwhelming event of a woman´s life, involving both medical interventions and expectations. The most important factors for a positive birth experience, as summarized by Hodnett et al. [30], are continuous support by midwives and women´s involvement in decision-making in their own labour, which was confirmed by studies included in this review [13, 44, 57, 65, 67]. If receiving pharmacological pain relief means less presence of midwives, it most likely affects the experience of childbirth negatively [86, 87].

One important factor having an impact on women´s expectations is their cultural environment [86, 88, 89].This cultural environment consists of previous birth experiences in her social circle, and what she believes is expected of her, from family and caregivers. Some women believe that expressions of pain might be viewed as a sign of weakness [1, 90]. An example of subtle influences is that the use of pharmacological pain-relief is not considered a part of natural birth [91]. Studies have looked at the connection between women´s expectations of their future labour and their actual experience of giving birth [32, 88, 92]. In many cases, the disappointments and dissatisfaction may be the result of a lack of realistic information regarding both levels of pain and of the effect of available pain relief [88, 93, 94]. Well-informed women with good support are more likely to be satisfied with their childbirth experience, with or without pharmacological pain relief. However, pain relief should be available to every labouring woman at request. Efforts should be made to avoid feelings of failure, regardless of what kind of pain relief the woman prefers [1, 86].

WHO´s recommendations for intrapartum care for a positive childbirth experience [1] emphasize safety, equity and cost effectiveness of antenatal interventions. Pain relief in obstetric care demands a substantial part of available anaesthetic services [95]. It is therefore important to ascertain that we prioritize our medical interventions correctly and evaluate outcomes [11, 15, 85].

### Strengths and limitations

A strength of our study was the thorough and systematic search, with repeated searches. The majority of data were retrieved from large cohort studies, including more than 15 000 women. In order to limit bias of the summarized data, we excluded studies not presenting multivariate regression analyses.

A limitation in our study was that we did not contact authors for missing data. Where no regression analyses were performed, authors might have supplied complementing data of relevant confounders to control for. More original data could have provided an opportunity to construct a more comprehensive forest plot. Further, no grey literature, pilot studies or unpublished papers were included. If these articles had presented a homogenous outcome, it could have effected our results. The language restriction, only including studies published in English, may have introduced a selection bias, potentially underrepresenting publications from non-Western and low-income communities. Further, in the construction of the search strategy, it is possible that we could have found more reports if other words or spellings had been included.

### Implications for future research

Well-conducted research, regarding when and how pain relief during labour should be offered for a positive birth experience, is warranted. Moreover, a ranking between different pain relief methods regarding their effect on satisfaction would be of clinical interest. Future studies need to be performed in a non-judgmental manner, directed towards a variety of populations. A core outcome set for labour pain management, a request previously raised in a systematic review by Tan [12], would improve the control of quality of care. Further, an agreement to use fewer standardized instruments for measuring birth experience would be valuable for comparisons of different populations and settings.

## Conclusion

This systematic review could not demonstrate an association between pharmacological pain relief and women´s experiences of childbirth, mainly due to large heterogeneity between studies and a lack of uniform assessment tools. We highlight the importance of defining and standardizing methods to measure birth satisfaction for a better comparability and an increasing understanding of how pharmacological pain relief is related to women´s birth experience.

High-quality research, with defined key outcomes, is urgently needed to correctly evaluate the effect of pain relief on overall birth satisfaction.

## Electronic supplementary material

Below is the link to the electronic supplementary material.

Supplementary Material 1

Supplementary Material 2

Supplementary Material 3

Supplementary Material 4

Supplementary Material 5

Supplementary Material 6

Supplementary Material 7

## Data Availability

All data generated or analysed during this study are included in this published article [and its supplementary information files].

## Abbreviations

CSE: Combined Spinal Epidural
GRADE: Grading and Recommendations, Assessment, Development and Evaluations
PRISMA: Preferred Reporting Items for Systematic Reviews and Meta-Analyses
PROSPERO: Prospective Register of Systematic Reviews
PTSD: Post-Traumatic Stress Disorder
Rayyan QCRI: AI powered tool for Systematic Reviews
RCT: Randomized Controlled Trials
RoB2: Risk of Bias 2 (Cochrane Collaboration tool)
ROBINS-I: Risk of Bias In Non-Randomized studies (Cochrane Collaboration tool)
Robvis: Risk of Bias VISualization tool
STATA 17: Statistical software for science
WHO: World Health Organization
