# Supplementary material for: Pharmacological pain relief and women´s birth experience: a systematic review
Source: BMC Pregnancy Childbirth. 2025 Apr 26;25:505. doi: 10.1186/s12884-025-07602-3 (PMC12032825; doi:10.1186/s12884-025-07602-3)
Supplement: Supplementary file 1 — Supplementary Material 1 [file 12884_2025_7602_MOESM1_ESM.docx]

**Additional file 3. Excluded articles with reasons**

| Study | Reason for exclusion |
| --- | --- |
| Abdolahian 2014^1^ | Inappropriate intervention. No pharmacological pain relief. |
| Ahmet 2019^2^ | Inappropriate outcome. No information of birth satisfaction |
| Akalin 2021^3^ | Inappropriate intervention. Mode of birth, not pharmacological pain relief. |
| Akin 2020^4^ | Inappropriate intervention. No pharmacological pain relief. |
| Aktas 2019^5^ | Inappropriate intervention. No information of pain relief. |
| Akköz 2021^6^ | Inappropriate intervention. No pharmacological pain relief. |
| Ali 2021^7^ | Inappropriate type of text. Conference abstract. |
| Al Shammari 2019^8^ | Inappropriate outcome. No information of overall birth satisfaction |
| Anim-Somuah 2011^9^ | Inappropriate type of text. A Systematic review |
| Anim-Somuah 2018^10^ | Inappropriate type of text. A Systematic review |
| Arendt 2013^11^ | Inappropriate type of text. Summary of previous studies. No information of pharmacological pain relief. |
| Babaoglu 2020^12^ | Inappropriate intervention. No information of pharmacological pain relief |
| Baez Suarez 2019^13^ | Inappropriate intervention. No information of pharmacological pain relief |
| Baljon 2022^14^ | Inappropriate intervention. No pharmacological pain relief. |
| Bernitz 2016^15^ | Inappropriate outcome. Satisfaction with care, not overall birth satisfaction. |
| Bhatt 2014^16^ | Inappropriate outcome. Birth satisfaction was related to what the women expected to experience, before they gave birth. |
| Boaviagem 2017^17^ | Inappropriate intervention. No pharmacological pain relief |
| Borup 2009^18^ | Inappropriate intervention and comparison. All participants received pharmacological pain relief. |
| Bricker ^19^ | Inappropriate type of text. A Systematic review |
| Britton 2012^20^ | Inappropriate type of text. A Systematic review |
| Bryanton 2008^21^ | Inappropriate intervention. The study looked at satisfaction with birth depending on mode of delivery. |
| Buehner 2011^22^ | Inappropriate outcome. No control group, all received Remifentanil-infusion |
| Burns 2000^23^ | Inappropriate intervention. No pharmacological pain relief. |
| Caballero 2016^24^ | Inappropriate intervention. No pharmacological pain relief. |
| Calik 2018^25^ | Unacceptable high risk of bias. No regression analysis made to control for confounders. |
| Chabbert 2021^26^ | Inappropriate type of text. A Systematic review |
| Chaillet 2014^27^ | Inappropriate type of text. A Systematic review |
| Chang 2002^28^ | Inappropriate intervention. No pharmacological pain relief. |
| Chang 2006^29^ | Inappropriate intervention. No pharmacological pain relief. |
| Cheng 2020^30^ | Inappropriate intervention. Information video, not pain relief. |
| Christiaens 2007^31^ | Inappropriate intervention. Pain, not pain relief |
| Cluett 2018^32^ | Inappropriate type of text. A Systematic review |
| Conesa Ferrer 2016^33^ | Inappropriate outcome. No validation of overall satisfaction. The intervention studied was level of care, in matters of a humanized versus a biomedical model. |
| Cooper 2010^34^ | Inappropriate outcome. Satisfaction with the pain relief method, not with overall birth experience. |
| Czech, 2018^35^ | Inappropriate outcome. Satisfaction with the pain relief method, not with overall birth experience. |
| Dahlen 2009^36^ | Inappropriate intervention. Warm packs for perineal pain was tested, no information about pharmacological pain relief. |
| Deepak 2022^37^ | Inappropriate outcome. No information of birth satisfaction. |
| Dencker 2010^38^ | Inappropriate intervention. No pharmacological pain relief. |
| Deshmukh 2018^39^ | Inappropriate outcome. Satisfaction with the pain relief method, not with overall birth experience. |
| Dickinson, 2002^40^ | Inappropriate outcome. VAS for pain measured, not overall satisfaction with birth. |
| Dickinson 2003^41^ | Inappropriate intervention and comparator. Both groups received different forms of pharmacological pain relief. |
| Dixon 2019^42^ | Inappropriate outcome. The aim was to study the time to request for analgesics, when receiving distraction by music or videogames. |
| Donate-Manzanares 2021^43^ | Inappropriate outcome. No information of overall birth satisfaction. |
| Dowswell 2009^44^ | Inappropriate type of text. A Systematic review |
| Downe 2015^45^ | Inappropriate outcome. Time to request for EDA, with versus without hypnosis. No information of overall birth satisfaction. |
| Duale 2014^46^ | Inappropriate type of text. A Systematic review |
| Ebrahimian 2019^47^ | Inappropriate intervention. No information of pain relief. |
| Erenoglu 2019^48^ | Inappropriate intervention. No information of pain relief. |
| Fernandes 2021^49^ | Inappropriate outcome. No information of overall birth satisfaction. |
| Floris 2017^50^ | Inappropriate outcome. Satisfaction in relation to expectations being fulfilled, not related to pain relief. |
| Fontein 2010^51^ | Inappropriate intervention. Birth satisfaction in relation to level of care was studied. No information of pain relief. |
| Gallo 2018^52^ | Inappropriate outcome. VAS for pain measured, not overall satisfaction with birth. |
| Gau 2011^53^ | Inappropriate outcome. VAS for pain measured, not overall satisfaction with birth. Both groups received pharmacological pain relief |
| Ghanbari-Homayi 2019^54^ | Inappropriate intervention. Looking at the option of pain relief, not pharmacological pain relief itself. |
| Ghanbari-Homaie 2021^55^ | Inappropriate intervention. Satisfaction related to degree of pain, not to pain relief. |
| Ghanbari-Homaie 2022^56^ | Inappropriate type of text. A Systematic review |
| Goodman 2004^57^ | Inappropriate intervention. Pharmacological and non-pharmacological pain relief methods were not separated. |
| Gregory 2019^58^ | Inappropriate intervention. Personal control, expectations and pain related to satisfaction was studied, not pain relief. |
| Gungor 2012^59^ | Inappropriate outcome. No validation of birth satisfaction. |
| Gupta 2020^60^ | Inappropriate outcome. No information of birth satisfaction. |
| GÖNenC 2020^61^ | Inappropriate intervention. Use of pharmacological pain relief was an exclusion criterion. |
| Hall 2020^62^ | Inappropriate outcome. The compared groups were inappropriate: both groups received pharmacological pain relief. |
| Halls 2008^63^ | Inappropriate intervention. No information of pharmacological pain relief. |
| Hauk 2019^64^ | Inappropriate intervention. No use of pharmacological pain relief |
| Henriksen 2017^65^ | Inappropriate outcome. No information of birth satisfaction in general. |
| Henry 2004^66^ | Inappropriate intervention. Pain, not pain relief |
| Hildingsson 2021^67^ | Unacceptable high risk of bias. No regression analyses performed to control for confounders. |
| Hitzeman 2012^68^ | Inappropriate type of text. A Systematic review |
| Hodnett 2002^69^ | Inappropriate type of text. A Systematic review |
| Hollins-Martin 2014^70^ | Inappropriate outcome. No validation of birth satisfaction. |
| Houser 2019^71^ | Inappropriate outcome. No validation of overall birth experience. |
| Howarth 2019^72^ | Inappropriate intervention. No use of pharmacological pain relief. |
| Howell 2001^73^ | Inappropriate intervention. Both groups received pharmacological pain relief. |
| Huang 2019^74^ | Inappropriate intervention. No information of pharmacological pain relief. |
| Ismail 2016^75^ | Inappropriate outcome. VAS for pain measured, not for overall birth experience. |
| Jafari 2017^76^ | Inappropriate outcome. Satisfaction with the pain relief method. No comparison of different groups of pain relief methods regarding satisfaction. |
| Jones 2012^77^ | Inappropriate type of text. A Systematic Review |
| Kannan 2001^78^ | Inappropriate outcome. Satisfaction with the pain relief method. No comparison of different groups of pain relief methods regarding satisfaction. |
| Kaplan 2021^79^ | Inappropriate intervention. No description of pharmacological pain relief. |
| Kapoor 2022^80^ | Inappropriate outcome. VAS for pain measured, not for overall birth experience. |
| Khaskheli 2010^81^ | Unacceptable high risk of bias. No regression analyses to control for confounders. |
| Khumalo 2020^82^ | Inappropriate outcome. No validation of overall birth experience. |
| Kortet 2021^83^ | Inappropriate outcome. No evaluation of birth satisfaction related to pain relief. |
| Labreque 1999^84^ | Inappropriate intervention. No validation of pharmacological pain relief. |
| Larkin 2017^85^ | Inappropriate outcome. No evaluation of birth satisfaction related to pain relief. |
| Lavender 1999^86^ | Inappropriate outcome. No evaluation of birth satisfaction related to pain relief. |
| Lee 2013^87^ | Inappropriate intervention. No validation of pharmacological pain relief. |
| Leeners 2016^88^ | Inappropriate outcome. Satisfaction with care, not with overall birth experience. |
| Lei 2015^89^ | Inappropriate type of text. Book chapter. |
| Leslie 2007^90^ | Inappropriate type of text. Book chapter. |
| Leung 2013^91^ | Inappropriate intervention. No validation of pharmacological pain relief. |
| Li 2012^92^ | Inappropriate language. Chinese. |
| Likis 2012^93^ | Inappropriate type of text. Book chapter |
| Likis 2014^94^ | Inappropriate type of text. A Systematic Review |
| Louvel 2021^95^ | Inappropriate outcome. VAS for pain measured, not for overall birth experience. |
| Macarthur 2010^96^ | Inappropriate outcome. No evaluation of birth satisfaction related to pain relief. |
| Madden 2016^97^ | Inappropriate type of text. A Systematic Review. |
| Maghalian 2021^98^ | Inappropriate intervention. No validation of pharmacological pain relief. |
| Maimburg 2016^99^ | Inappropriate intervention. No information of pharmacological pain relief. |
| Mahmohdikuani 2019^100^ | Inappropriate intervention. No comparison of different groups of pain relief methods regarding satisfaction. |
| Mansoori, 2000^101^ | Inappropriate outcome. Only effect of pain relief, no validation of overall birth satisfaction. |
| Martin 2011^102^ | Inappropriate outcome. Satisfaction with pain relief method, no overall birth satisfaction. |
| Miyakoshi 2013^103^ | Inappropriate outcome. Construction of a questionnaire. No evaluation of birth satisfaction. |
| Mortazavi 2022^104^ | Inappropriate type of text. Pre print only. |
| Moschini 2006^105^ | Inappropriate outcome. Satisfaction with pain relief method, no overall birth satisfaction. |
| Moudi 2016^106^ | Inappropriate outcome. No validation of overall birth satisfaction. |
| Mousavi 2020^107^ | Inappropriate outcome. Satisfaction with the pain relief method. No information of different groups of pain relief methods related to overall birth satisfaction. |
| Mokhopadhyay 2022^108^ | Inappropriate outcome. PTSD, not overall satisfaction with birth. |
| Mutabazi 2021^109^ | Inappropriate outcome. Satisfaction with the pain relief method, not overall satisfaction with birth. |
| Nesheim 2003^110^ | Inappropriate outcome. VAS for pain measured, not for overall birth experience. |
| Newnham 2021^111^ | Inappropriate outcome. Satisfaction with the pain relief method, not overall satisfaction with birth. Both groups received pharmacological analgesia. |
| Nikodem 2022^112^ | Inappropriate intervention. No pharmacological pain relief. |
| Novikova 2012^113^ | Inappropriate type of text. A Systematic Review |
| Nutter 2014^114^ | Inappropriate type of text. A Peer Review |
| Nystedt 2005^115^ | Inappropriate intervention. No information of pharmacological pain relief |
| Ortiz-Contreras 2021^116^ | Inappropriate intervention. No pharmacological pain relief. |
| [O'Sullivan](javascript:searchAuthor('O\'Sullivan,%20G.')) 2008^117^ | Inappropriate type of text. Refresher text. Letter to Editor. |
| Othman 2012^118^ | Inappropriate type of text. Narrative review. |
| Oweis 2009^119^ | Inappropriate intervention. Information of pain, not of pain relief |
| Ozkan 2019^120^ | Inappropriate population. Cesarean Section and vaginal birth were not reported separately. |
| [Pasha](javascript:searchAuthor('Pasha,%20H.')) 2012^121^ | Inappropriate outcome. Satisfaction with the pain relief method, not with overall birth experience. |
| Pasha 2012^122^ | Inappropriate outcome. Satisfaction with the pain relief method, not with overall birth experience. |
| Pennell 2011^123^ | Inappropriate outcome. Satisfaction with birth plan, not with overall birth experience. |
| Pietrzak 2022^124^ | Inappropriate outcome. Satisfaction with pain relief methods, no information of overall birth satisfaction. |
| Pozo-Cano 2020^125^ | Inappropriate outcome. No validation of overall birth satisfaction. Primary aim to validate a questionnaire. |
| Qian 2018^126^ | Inappropriate outcome. No information of overall birth satisfaction. |
| Rahimi-Kian 2018^127^ | Inappropriate intervention. No pharmacological pain relief. |
| Ramnerö 2002^128^ | Inappropriate outcome. Validation of pain relief methods, not of overall birth satisfaction.. |
| [Rao](javascript:searchAuthor('Rao,%20Z.%20A.')) 2010^129^ | Inappropriate outcome. Satisfaction with the pain relief method, not overall birth experience. Both groups received pharmacological analgesia. |
| Redshaw 2009^130^ | Inappropriate outcome. No validation of overall birth satisfaction. |
| Redshaw 2019^131^ | Inappropriate outcome. No validation of overall birth satisfaction. |
| Richardsson 2016^132^ | Inappropriate type of text. A historical review. |
| [Rolinska](javascript:searchAuthor('Rolinska,%20A.%20A.')) 2015^133^ | Inappropriate outcome. VAS for pain measured, not overall birth satisfaction. |
| Romero Gonzalez 2019^134^ | Inappropriate intervention. Both groups received pharmacological analgesia. |
| Salim 2005^135^ | Inappropriate outcome. No validation of overall birth satisfaction. Both groups received pharmacological pain relief. |
| Santana 2016^136^ | Inappropriate intervention. Both groups received pharmacological analgesia. |
| Sawyer 2014^137^ | Inappropriate outcome. No validation of overall birth satisfaction. Validation of questionnaire. |
| Shaban 2016^138^ | Inappropriate outcome. No validation of overall birth satisfaction. Validation of questionnaire. |
| Shapiro 1998^139^ | Inappropriate outcome. Satisfaction with care. Both groups received pharmacological pain relief. |
| Shaw-Battista 2017^140^ | Inappropriate type of text. A Systematic Review. |
| Shivanagappa 2021^141^ | Inappropriate outcome. Satisfaction with the pain relief method, not overall satisfaction |
| Simavli 2014^142^ | Inappropriate intervention. No information of pharmacological pain relief.  . |
| Simkin 2002^143^ | Inappropriate type of text. A Systematic Review. |
| Simkin 2004^144^ | Inappropriate type of text. A narrative Review. |
| Skodova 2019^145^ | Inappropriate type of text. A narrative Review. |
| Smith 2006^146^ | Inappropriate type of text. A Systematic Review. |
| Smith 2018^147^ | Inappropriate type of text. A Systematic Review. |
| Smith 2018^148^ | Inappropriate type of text. A Systematic Review. |
| Smith 2018^149^ | Inappropriate type of text. A Systematic Review. |
| [Stadlmayr](javascript:searchAuthor('Stadlmayr,%20W.')) 2004^150^ | Inappropriate outcome. No validation of overall birth experience |
| Sulmi 2021^151^ | Inappropriate outcome. No validation of overall birth satisfaction. Primary aim to describe misperceptions regarding epidural analgesia. |
| Taavoni 2013^152^ | Inappropriate intervention. No information of pharmacological pain relief. |
| Taheri 2018^153^ | Inappropriate intervention. Both groups received the same pain relief. No information of pharmacological pain relief. |
| Thomson 2019^154^ | Inappropriate type of text. A Systematic Review. |
| Thuvrakan ^155^ 2020 | Inappropriate type of text. Qualitative systematic review |
| Tiznobaik 2019^156^ | Unacceptable risk of bias due to methodological uncertainties. |
| Tournaire 2007^157^ | Inappropriate type of text. A Systematic Review. |
| Tsui 2004^158^ | Inappropriate outcome. Satisfaction with pain relief method, not with overall birth experience.  . |
| Ulfsdottir 2019^159^ | Inappropriate intervention. Water birth, not pharmacological pain relief. |
| Ullman 2010^160^ | Inappropriate type of text. A Systematic Review.. |
| Unalmis Erdogan 2017^161^ | Inappropriate type of text. A Systematic Review. |
| van der Spank 2000^162^ | Inappropriate intervention. Ni information of pharmacological pain relief. |
| Van Stenus 2018^163^ | Inappropriate outcome. Incidence or request for epidural analgesia with TENS as compared without. |
| Vixner 2015^164^ | Inappropriate outcome. VAS for pain relief measured, not for overall birth experience. |
| Waldenstrom 2000^165^ | Inappropriate outcome. Satisfaction with healthcare, not with general birth experience. |
| Walker 2015^166^ | Inappropriate outcome. No validation of overall birth satisfaction. Validation of questionnaire. |
| Weng 2023^167^ | Inappropriate outcome. Satisfaction with sense of control, not overall birth experience. |
| Weibel 2017^168^ | Inappropriate type of text. A Systematic Review. |
| Westergren 2021^169^ | Unacceptable high risk of bias. No information of accuracy of data. |
| Wilde-Larsson 2010^170^ | Inappropriate outcome. No validation of overall birth satisfaction. Validation of questionnaire. |
| Yeung 2019^171^ | Inappropriate type of text. Study protocol |
| Xing 2015^172^ | Inappropriate outcome. No validation of overall birth satisfaction. |
| [Yildirim](javascript:searchAuthor('Yildirim,%20G.')) 2004^173^ | Inappropriate outcome. Only validation of VAS for pain relief, not for overall birth satisfaction. |
| Yurashevich 2019^174^ | Inappropriate intervention. No information of pharmacological pain relief. |
| Zhu 2019^175^ | Unacceptable high risk of bias. No regression analysis to control for confounders. |

1. Abdolahian S, Ghavi F, Abdollahifard S, Sheikhan F. Effect of dance labor on the management of active phase labor pain & clients' satisfaction: a randomized controlled trial study. *Glob J Health Sci* 2014: **6**: 219-26.

2. Ahmet G, Ebru C. Epidural analgesia for labor pain: What has changed in the last 1 year? Literature review and clinical results our experience in labor analgesia: Labor analgesia effectiveness. *Journal of Clinical and Analytical Medicine* 2019: **10**: 135-41.

3. Akalin A, Sahin S. Nulliparous Women's Birth Perceptions and Experiences for Mode of Birth Preference: A Qualitative Descriptive Study. *International Journal of Caring Sciences* 2021: **14**: 1000-8.

4. Akin B, Saydam BK. The effect of labor dance on perceived labor pain, birth satisfaction, and neonatal outcomes. *Explore (NY)* 2020: **16**: 310-7.

5. Aktaş S, Aydın R. The analysis of negative birth experiences of mothers: a qualitative study. *J Reprod Infant Psychol* 2019: **37**: 176-92.

6. Akköz Çevik S, Incedal İ. The effect of reflexology on labor pain, anxiety, labor duration, and birth satisfaction in primiparous pregnant women: a randomized controlled trial. *Health Care Women Int* 2021: **42**: 710‐25.

7. Ali RM. Assessment of women’s satisfaction with childbirth experience after utilization of pain management practices at Al-Elwyia Maternity Teaching Hospital. *Medico-Legal Update* 2021: **21**: 940-3.

8. Al-Shammari I, Roa L, Yorlets RR, Akerman C, Dekker A, Kelley T, et al. Implementation of an international standardized set of outcome indicators in pregnancy and childbirth in Kenya: Utilizing mobile technology to collect patient-reported outcomes. *PLoS One* 2019: **14**.

9. Anim-Somuah M, Smyth RM, Jones L. Epidural versus non-epidural or no analgesia in labour. *Cochrane Database Syst Rev* 2011: Cd000331.

10. Anim‐Somuah M, Smyth RMD, Cyna AM, Cuthbert A. Epidural versus non‐epidural or no analgesia for pain management in labour. *Cochrane Database Syst Rev* 2018.

11. Arendt KW, Tessmer-Tuck JA. Nonpharmacologic labor analgesia. *Clin Perinatol* 2013: **40**: 351-71.

12. Babaoğlu G, Kiliçaslan B, Ankay Yilbaş A, Çelebioğlu B. Effects of different analgesic methods used for vaginal delivery on mothers and fetuses. *Turk J Med Sci* 2020: **50**: 930-6.

13. Baez Suarez A, Martin Castillo E, Garcia Andujar J, Garcia Hernandez JA, Quintana Montesdeoca MP, Loro Ferrer JF. Evaluation of the effectiveness of transcutaneous nerve stimulation during labor in breech presentation: a case series. *J Matern Fetal Neonatal Med* 2019.

14. Baljon K, Romli MH, Ismail AH, Khuan L, Chew BH. Effectiveness of Breathing Exercises, Foot Reflexology and Massage (BRM) on Maternal and Newborn Outcomes Among Primigravidae in Saudi Arabia: A Randomized Controlled Trial. *Int J Womens Health* 2022: **14**: 279-95.

15. Bernitz S, Oian P, Sandvik L, Blix E. Evaluation of satisfaction with care in a midwifery unit and an obstetric unit: a randomized controlled trial of low-risk women. *BMC Pregnancy Childbirth* 2016: **16**: 143.

16. Bhatt H, Pandya S, Kolar G, Nirmalan PK. The impact of labour epidural analgesia on the childbirth expectation and experience at a tertiary care center in southern India. *Journal of Clinical and Diagnostic Research* 2014: **8**: 73-6.

17. Boaviagem A, Melo Junior E, Lubambo L, Sousa P, Aragao C, Albuquerque S, et al. The effectiveness of breathing patterns to control maternal anxiety during the first period of labor: A randomized controlled clinical trial. *Complement Ther Clin Pract* 2017: **26**: 30-5.

18. Borup L, Wurlitzer W, Hedegaard M, Kesmodel US, Hvidman L. Acupuncture as pain relief during delivery: a randomized controlled trial. *Birth* 2009: **36**: 5-12.

19. Bricker L, Lavender T. Parenteral opioids for labor pain relief: A systematic review. *Am J Obstet Gynecol* 2002: **186**: S94-S109.

20. Britton JR. The assessment of satisfaction with care in the perinatal period. *Journal of Psychosomatic Obstetrics and Gynecology* 2012: **33**: 37-44.

21. Bryanton J, Gagnon AJ, Johnston C, Hatem M. Predictors of women's perceptions of the childbirth experience. *J Obstet Gynecol Neonatal Nurs* 2008: **37**: 24-34.

22. Buehner U, Broadbent JR, Chesterfield B. Remifentanil patient-controlled analgesia for labour: a complete audit cycle. *Anaesth Intensive Care* 2011: **39**: 666-70.

23. Burns EE, Blamey C, Ersser SJ, Barnetson L, Lloyd AJ. An investigation into use of aromatherapy in intrapartum midwifery practice. *J Altern Complement Med* 2000: **6**: 141-7.

24. Caballero P, Delgado-Garcia BE, Orts-Cortes I, Moncho J, Pereyra-Zamora P, Nolasco A. Validation of the Spanish version of Mackey childbirth satisfaction rating scale. *BMC Pregnancy Childbirth* 2016: **16**: 78.

25. Calik KY, Karabulutlu O, Yavuz C. First do no harm - interventions during labor and maternal satisfaction: a descriptive cross-sectional study. *BMC Pregnancy Childbirth* 2018: **18**: 415.

26. Chabbert M, Panagiotou D, Wendland J. Predictive factors of women's subjective perception of childbirth experience: a systematic review of the literature. *J Reprod Infant Psychol* 2021: **39**: 43-66.

27. Chaillet N, Belaid L, Crochetiere C, Roy L, Gagne GP, Moutquin JM, et al. Nonpharmacologic approaches for pain management during labor compared with usual care: a meta-analysis. *Birth* 2014: **41**: 122-37.

28. Chang MY, Wang SY, Chen CH. Effects of massage on pain and anxiety during labour: a randomized controlled trial in Taiwan. *J Adv Nurs* 2002: **38**: 68-73.

29. Chang MY, Chen CH, Huang KF. A comparison of massage effects on labor pain using the McGill Pain Questionnaire. *J Nurs Res* 2006: **14**: 190-7.

30. Cheng WJ, Hung KC, Ho CH, Yu CH, Chen YC, Wu MP, et al. Satisfaction in parturients receiving epidural analgesia after prenatal shared decision-making intervention: a prospective, before-and-after cohort study. *BMC Pregnancy Childbirth* 2020: **20**: 413.

31. Christiaens W, Bracke P. Assessment of social psychological determinants of satisfaction with childbirth in a cross-national perspective. *BMC Pregnancy Childbirth* 2007: **7**: 26.

32. Cluett ER, Burns E, Cuthbert A. Immersion in water during labour and birth. *Cochrane Database Syst Rev* 2018: **5**: Cd000111.

33. Conesa Ferrer MB, Canteras Jordana M, Ballesteros Meseguer C, Carrillo Garcia C, Martinez Roche ME. Comparative study analysing women's childbirth satisfaction and obstetric outcomes across two different models of maternity care. *BMJ Open* 2016: **6**: e011362.

34. Cooper GM, MacArthur C, Wilson MJ, Moore PA, Shennan A. Satisfaction, control and pain relief: short- and long-term assessments in a randomised controlled trial of low-dose and traditional epidurals and a non-epidural comparison group. *Int J Obstet Anesth* 2010: **19**: 31-7.

35. Czech I, Fuchs P, Fuchs A, Lorek M, Tobolska-Lorek D, Drosdzol-Cop A, et al. Pharmacological and non-pharmacological methods of labour pain relief—establishment of effectiveness and comparison. *Int J Environ Res Public Health* 2018: **15**.

36. Dahlen HG, Homer CS, Cooke M, Upton AM, Nunn RA, Brodrick BS. 'Soothing the ring of fire': Australian women's and midwives' experiences of using perineal warm packs in the second stage of labour. *Midwifery* 2009: **25**: e39-48.

37. Deepak D, Kumari A, Mohanty R, Prakash J, Kumar T, Priye S. Effects of Epidural Analgesia on Labor Pain and Course of Labor in Primigravid Parturients: A Prospective Non-randomized Comparative Study. *Cureus* 2022: **14**: e26090.

38. Dencker A, Taft C, Bergqvist L, Lilja H, Berg M. Childbirth experience questionnaire (CEQ): development and evaluation of a multidimensional instrument. *BMC Pregnancy Childbirth* 2010: **10**: 81.

39. Deshmukh VL, Ghosh SS, Yelikar KA, Gadappa SN. Effects of Epidural Labour Analgesia in Mother and Foetus. *J Obstet Gynaecol India* 2018: **68**: 111-6.

40. Dickinson JE, Paech MJ, McDonald SJ, Evans SF. The impact of intrapartum analgesia on labour and delivery outcomes in nulliparous women. *Aust N Z J Obstet Gynaecol* 2002: **42**: 59-66.

41. Dickinson JE, Paech MJ, McDonald SJ, Evans SF. Maternal satisfaction with childbirth and intrapartum analgesia in nulliparous labour. *Aust N Z J Obstet Gynaecol* 2003: **43**: 463-8.

42. Dixon CL, Monsivais L, Chamseddine P, Olson G, Pacheco LD, Saade GR, et al. The Effect of Distraction during Labor Induction on Timing of Analgesia Request: A Randomized Clinical Trial. *Am J Perinatol* 2019: **36**: 1351-6.

43. Donate‐Manzanares M, Rodríguez‐Cano T, Rodríguez‐Almagro J, Hernández‐Martínez A, Santos‐Hernández G, Beato‐Fernández L. Mixed‐method study of women's assessment and experience of childbirth care. *Journal of Advanced Nursing (John Wiley & Sons, Inc)* 2021: **77**: 4195-210.

44. Dowswell T, Bedwell C, Lavender T, Neilson JP. Transcutaneous electrical nerve stimulation (TENS) for pain relief in labour. *Cochrane Database Syst Rev* 2009.

45. Downe S, Finlayson K, Melvin C, Spiby H, Ali S, Diggle P, et al. Self-hypnosis for intrapartum pain management in pregnant nulliparous women: a randomised controlled trial of clinical effectiveness. *BJOG* 2015: **122**: 1226-34.

46. Duale C, Nicolas-Courbon A, Gerbaud L, Lemery D, Bonnin M, Pereira B. Maternal satisfaction as an outcome criterion in research on labor analgesia: data analysis from the recent literature. *Clin J Pain* 2015: **31**: 235-46.

47. Ebrahimian A, Rahmani Bilandi R. Comparisons of the Effects of Watching Virtual Reality Videos and Chewing Gum on the Length of Delivery Stages and Maternal Childbirth Satisfaction: a Randomized Controlled Trial. *Iranian journal of medical sciences* 2021: **46**: 15‐22.

48. Erenoglu R, Baser M. Effect of expressıve touchıng on labour paın and maternal satısfactıon: a randomized controlled trial. *Complement Ther Clin Pract* 2019: **34**: 268‐74.

49. Fernandes S, Galacho J, Borrego A, Pereira D, Lança F, Ormonde L. Impact of Labor Epidural Analgesia on Maternal Satisfaction and Childbirth Expectations in a Tertiary Care Center in Portugal: A Prospective Study. *Acta Med Port* 2021: **34**: 272-7.

50. Floris L, Irion O, Courvoisier D. Influence of obstetrical events on satisfaction and anxiety during childbirth: a prospective longitudinal study. *Psychol Health Med* 2017: **22**: 969-77.

51. Fontein Y. The comparison of birth outcomes and birth experiences of low-risk women in different sized midwifery practices in the Netherlands. *Women Birth* 2010: **23**: 103-10.

52. Gallo RBS, Santana LS, Marcolin AC, Duarte G, Quintana SM. Sequential application of non-pharmacological interventions reduces the severity of labour pain, delays use of pharmacological analgesia, and improves some obstetric outcomes: a randomised trial. *J Physiother* 2018: **64**: 33-40.

53. Gau ML, Chang CY, Tian SH, Lin KC. Effects of birth ball exercise on pain and self-efficacy during childbirth: a randomised controlled trial in Taiwan. *Midwifery* 2011: **27**: e293-300.

54. Ghanbari-Homayi S, Fardiazar Z, Meedya S, Mohammad-Alizadeh-Charandabi S, Asghari-Jafarabadi M, Mohammadi E, et al. Predictors of traumatic birth experience among a group of Iranian primipara women: a cross sectional study. *BMC Pregnancy Childbirth* 2019: **19**: N.PAG-N.PAG.

55. Ghanbari-Homaie S, Meedya S, Mohammad-Alizadeh-Charandabi S, Jafarabadi MA, Mohammadi E, Mirghafourvand M. Recommendations for improving primiparous women's childbirth experience: results from a multiphase study in Iran. *Reprod Health* 2021: **18**: 146.

56. Ghanbari-Homaie S, Hasani S, Pourfathi H, Mirghafourvand M. Effectiveness of Pharmacological Pain Relief Methods on Birth Experience: A Systematic Review and Meta-Analysis. *Current Women's Health Reviews* 2022: **18**: 61-9.

57. Goodman P, Mackey MC, Tavakoli AS. Factors related to childbirth satisfaction. *J Adv Nurs* 2004: **46**: 212-9.

58. Gregory KD, Korst LM, Saeb S, McCulloch J, Greene N, Fink A, et al. Childbirth-specific patient-reported outcomes as predictors of hospital satisfaction. *Am J Obstet Gynecol* 2019: **220**: 201.e1-.e19.

59. Gungor I, Beji NK. Development and psychometric testing of the scales for measuring maternal satisfaction in normal and caesarean birth. *Midwifery* 2012: **28**: 348-57.

60. Gupta R, Kaur G, Kaur J, Chawla S, Kaur S, Kullar K, et al. Evaluating the effectiveness of TENS for maternal satisfaction in laboring parturients - Comparison with epidural analgesia. *Journal of Anaesthesiology Clinical Pharmacology* 2020: **36**: 500-5.

61. GÖNenÇ IM, TerzioĞLu F. Effects of Massage and Acupressure on Relieving Labor Pain, Reducing Labor Time, and Increasing Delivery Satisfaction. *Journal of Nursing Research (Lippincott Williams & Wilkins)* 2020: **28**: e68-e.

62. Hall PJ, Foster JW, Yount KM, Jennings BM. Comfort in Labor: "Like Being Able to Exhale". *The Journal of perinatal & neonatal nursing* 2020: **34**: 38-45.

63. Halls KL. Maternal satisfaction regarding anaesthetic services during childbirth. *British Journal of Midwifery* 2008: **16**: 296-301.

64. Hauck Y, Lewis L, Overing H, Poletti C, Barnes C, Black N, et al. Research Article Women's experience of maternity high-dependency care following a complicated birth: A cross-sectional study. *Intensive Crit Care Nurs* 2019: **53**: 54-9.

65. Henriksen L, Grimsrud E, Schei B, Lukasse M. Factors related to a negative birth experience - A mixed methods study. *Midwifery* 2017: **51**: 33-9.

66. Henry A, Nand SL. Intrapartum pain management at the Royal Hospital for Women. *Aust N Z J Obstet Gynaecol* 2004: **44**: 307-13.

67. Hildingsson I, Karlström A, Larsson B. Childbirth experience in women participating in a continuity of midwifery care project. *Women and Birth* 2021: **34**: e255-e61.

68. Hitzeman N, Chin S. Epidural analgesia for labor pain. *Am Fam Physician* 2012: **86**: 241-2.

69. Hodnett ED. Pain and women's satisfaction with the experience of childbirth: a systematic review. *Am J Obstet Gynecol* 2002: **186**: S160-72.

70. Hollins Martin CJ, Martin CR. Development and psychometric properties of the Birth Satisfaction Scale-Revised (BSS-R). *Midwifery* 2014: **30**: 610-9.

71. Houser T, DeButy K, Beal CC. Implementation of an Evidence-Based Practice Change to Offer Nitrous Oxide During Labor. *Nurs Womens Health* 2019: **23**: 11-20.

72. Howarth AM, Swain NR. Low-cost, self-paced, educational programmes increase birth satisfaction in first-time mothers. *New zealand college of midwives journal* 2019: 14‐9.

73. Howell CJ, Kidd C, Roberts W, Upton P, Lucking L, Jones PW, et al. A randomised controlled trial of epidural compared with non-epidural analgesia in labour. *BJOG* 2001: **108**: 27-33.

74. Huang D, Dai L, Zeng T, Huang H, Wu M, Yuan M, et al. Exploring Contributing Factors to Psychological Traumatic Childbirth from the Perspective of Midwives: A Qualitative Study. *Asian Nurs Res (Korean Soc Nurs Sci)* 2019: **13**: 270-6.

75. Ismail S, Abbasi S, Khan S, Monem A, Afshan G. Factors associated with choice of delivery with or without epidural analgesia among laboring women: a cross-sectional survey at a tertiary care hospital of a developing country. *J Perinat Med* 2016: **44**: 799-806.

76. Jafari E, Mohebbi P, Mazloomzadeh S. Factors Related to Women's Childbirth Satisfaction in Physiologic and Routine Childbirth Groups. *Iran J Nurs Midwifery Res* 2017: **22**: 219-24.

77. Jones L, Othman M, Dowswell T, Alfirevic Z, Gates S, Newburn M, et al. Pain management for women in labour: an overview of systematic reviews. *Cochrane Database Syst Rev* 2012: Cd009234.

78. Kannan S, Jamison RN, Datta S. Maternal satisfaction and pain control in women electing natural childbirth. *Reg Anesth Pain Med* 2001: **26**: 468-72.

79. Kaplan E, Çevik S. The effect of guided imagery and reflexology on pain intensity, duration of labor and birth satisfaction in primiparas: randomized controlled trial. *Health Care Women Int* 2021: **42**: 691‐709.

80. Kapoor NB, Bagdi M. Influence of Epidural Analgesia on Pain Relief, Progression of Labour and Neonatal Outcome: A Comparative Study. *International Journal of Pharmaceutical and Clinical Research* 2022: **14**: 218-25.

81. Khaskheli M, Baloch S. Subjective pain perceptions during labour and its management. *J Pak Med Assoc* 2010: **60**: 473-6.

82. Khumalo N, Rwakaikara E. Patient satisfaction with peri-partum care at Bertha Gxowa district hospital, South Africa. *African journal of primary health care & family medicine* 2020: **12**: e1-e8.

83. Kortet S, Melender H-L, Klemetti R, Kääriäinen M, Kaakinen P. Mothers' perceptions of the quality of maternity services at Finnish maternity units: A cross-sectional study. *Nordic Journal of Nursing Research* 2021: **41**: 14-24.

84. Labrecque M, Nouwen A, Bergeron M, Rancourt JF. A randomized controlled trial of nonpharmacologic approaches for relief of low back pain during labor. *J Fam Pract* 1999: **48**: 259-63.

85. Larkin P, Begley CM, Devane D. Women's preferences for childbirth experiences in the Republic of Ireland; a mixed methods study. *BMC Pregnancy Childbirth* 2017: **17**: 19.

86. Lavender T, Walkinshaw SA, Walton I. A prospective study of women's views of factors contributing to a positive birth experience. *Midwifery* 1999: **15**: 40-6.

87. Lee SL, Liu CY, Lu YY, Gau ML. Efficacy of warm showers on labor pain and birth experiences during the first labor stage. *J Obstet Gynecol Neonatal Nurs* 2013: **42**: 19-28.

88. Leeners B, Görres G, Block E, Hengartner MP. Birth experiences in adult women with a history of childhood sexual abuse. *J Psychosom Res* 2016: **83**: 27-32.

89. Lei FQ, Feng C, Zhou Q. Effect of remifentanil-leading multidimensional labor analgesia on neonatal apgar score and the analgesic effect. *Maternal and child health care of china [zhong guo fu you bao jian]* 2015: **30**: 2771‐3.

90. Leslie MS, Romano A, Woolley D. Step 7: educates staff in nondrug methods of pain relief and does not promote use of analgesic, anesthetic drugs: the coalition for improving maternity services. *J Perinat Educ* 2007: **16 Suppl 1**: 65s-73s.

91. Leung RW, Li JF, Leung MK, Fung BK, Fung LC, Tai SM, et al. Efficacy of birth ball exercises on labour pain management. *Hong Kong Med J* 2013: **19**: 393-9.

92. Li HB, Liu ZQ, Chen XB, Ma XX, Huan Y. Clinical efficacy of diverse intravertebral infusion with sufentanil and ropivacaine for labor analgesia. *Journal of shanghai jiaotong university (medical science)* 2012: **32**: 499‐502.

93. Likis FE, Andrews JC, Collins MR, Lewis RM, Seroogy JJ, Starr SA, et al. AHRQ Comparative Effectiveness Reviews. *Nitrous Oxide for the Management of Labor Pain*. Rockville (MD): Agency for Healthcare Research and Quality (US); 2012.

94. Likis FE, Andrews JC, Collins MR, Lewis RM, Seroogy JJ, Starr SA, et al. Nitrous oxide for the management of labor pain: a systematic review. *Anesth Analg* 2014: **118**: 153-67.

95. Louvel L, Lorrain S, von Theobald P. Satisfaction of patients in pain management delivering in Reunion Island with or without neuraxial analgesia. *J Gynecol Obstet Hum Reprod* 2021: **50**: 102106.

96. Macarthur A, Imarengiaye C, Tureanu L, Downey K. A randomized, double-blind, placebo-controlled trial of epidural morphine analgesia after vaginal delivery. *Anesth Analg* 2010: **110**: 159-64.

97. Madden K, Middleton P, Cyna AM, Matthewson M, Jones L. Hypnosis for pain management during labour and childbirth. *Cochrane Database Syst Rev* 2016: Cd009356.

98. Maghalian M, Mirghafourvand M, Ghaderi F, Abbasalizadeh S, Pak S, kamalifard M. Comparison the effect of Swedish massage and interferential electrical stimulation on labor pain and childbirth experience in primiparous women: a randomized controlled clinical trial. *Arch Gynecol Obstet* 2022: **306**: 37-47.

99. Maimburg RD, Vaeth M, Dahlen H. Women's experience of childbirth - A five year follow-up of the randomised controlled trial "Ready for Child Trial". *Women Birth* 2016: **29**: 450-4.

100. Mahmoudikohani F, Torkzahrani S, Saatchi K, Nasiri M. Effects of acupressure on the childbirth satisfaction and experience of birth: a randomized controlled trial. *J Bodyw Mov Ther* 2019: **23**: 728‐32.

101. Mansoori S, Adams S, Cheater FM. Choice of analgesia in labour on neonatal outcomes, delivery and maternal satisfaction with pain relief. *Clin Eff Nurs* 2000: **4**: 11-9.

102. Martin CH, Fleming V. The birth satisfaction scale. *Int J Health Care Qual Assur* 2011: **24**: 124-35.

103. Miyakoshi K, Tanaka M, Morisaki H, Kim SH, Hosokawa Y, Matsumoto T, et al. Perinatal outcomes: intravenous patient-controlled fentanyl versus no analgesia in labor. *J Obstet Gynaecol Res* 2013: **39**: 783-9.

104. Mortazavi F, Mehrabadi M. A cross-sectional study of low birth satisfaction among Iranian postpartum women during COVID-19 epidemics’ fifth wave. 2022.

105. Moschini V, Marra G, Dabrowska D. Complications of epidural and combined spinal-epidural analgesia in labour. *Minerva Anestesiol* 2006: **72**: 47-58.

106. Moudi Z, Tavousi M. Evaluation of Mackey Childbirth Satisfaction Rating Scale in Iran: What Are the Psychometric Properties? *Nursing and Midwifery Studies* 2016: **5**: e29952.

107. Mousavi S, Nourizadeh R, Mokhtari F, Hakimi S, Babapour J, Mousavi S. Determinants of postpartum post-traumatic stress disorder: A cross-sectional study. *Crescent Journal of Medical and Biological Sciences* 2020: **7**: 254-9.

108. Mukhopadhyay I, Pruthvi Raj V, Aggarwal R. A randomized controlled trial of entonox and oxygen in labor analgesia. *Journal of SAFOG* 2021: **13**: 101‐5.

109. Mutabazi UP, Brysiewicz P. Descriptive survey of women's childbirth experiences in two state hospitals in KwaZulu-Natal. *Curationis* 2021: **44**: 1-7.

110. Nesheim BI, Kinge R, Berg B, Alfredsson B, Allgot E, Hove G, et al. Acupuncture during labor can reduce the use of meperidine: a controlled clinical study. *Clin J Pain* 2003: **19**: 187-91.

111. Newnham EC, Moran PS, Begley CM, Carroll M, Daly D. Comparison of labour and birth outcomes between nulliparous women who used epidural analgesia in labour and those who did not: A prospective cohort study. *Women and Birth* 2021: **34**: e435-e41.

112. Nikodem VC, Edwards SE, Krzyzanski AM, Berghella V, Hofmeyr GJ. Immersion in water during the second stage of labor: a randomized controlled trial. *Am J Obstet Gynecol MFM* 2022: **4**: 100721.

113. Novikova N, Cluver C. Local anaesthetic nerve block for pain management in labour. *Cochrane Database Syst Rev* 2012: N.PAG-N.PAG.

114. Nutter E, Meyer S, Shaw-Battista J, Marowitz A. Waterbirth: an integrative analysis of peer-reviewed literature. *J Midwifery Womens Health* 2014: **59**: 286-319.

115. Nystedt A, Hogberg U, Lundman B. The negative birth experience of prolonged labour: a case-referent study. *J Clin Nurs* 2005: **14**: 579-86.

116. Ortiz Contreras J, Adrián C, Fernández C, Mella M, Villagrán M, Diaz M, et al. Childbirth experiences of immigrant women in Chile: Trading human rights and autonomy for dignity and good care. *Midwifery* 2021: **101**: N.PAG-N.PAG.

117. O'Sullivan G. Non-neuraxial analgesia in labour. *Southern African Journal of Anaesthesia and Analgesia* 2008: **14**: 98-100.

118. Othman M, Jones L, Neilson JP. Non-opioid drugs for pain management in labour. *Cochrane Database Syst Rev* 2012: N.PAG-N.PAG.

119. Oweis A. Jordanian mother's report of their childbirth experience: findings from a questionnaire survey. *Int J Nurs Pract* 2009: **15**: 525-33.

120. Ozkan SA, Bal MD. Maternal Satisfaction in Normal and Caesarean Birth: A Cross-Sectional Study. *International Journal of Caring Sciences* 2019: **12**: 408-15.

121. Pasha H, Basirat Z, Hajiahmadi M, Bakhtiari A, Faramarzi M, Salmalian H. Evaluation of painless labor with entonox gas. *Journal of babol university of medical sciences* 2012: **14**: 71‐6.

122. Pasha H, Basirat Z, Hajahmadi M, Bakhtiari A, Faramarzi M, Salmalian H. Maternal expectations and experiences of labor analgesia with nitrous oxide. *Iran Red Crescent Med J* 2012: **14**: 792-7.

123. Pennell A, Salo-Coombs V, Herring A, Spielman F, Fecho K. Anesthesia and Analgesia-Related Preferences and Outcomes of Women Who Have Birth Plans. *Journal of Midwifery & Women's Health* 2011: **56**: 376-81.

124. Pietrzak J, Mędrzycka-Dąbrowska W, Tomaszek L, Grzybowska ME. A Cross-Sectional Survey of Labor Pain Control and Women's Satisfaction. *Int J Environ Res Public Health* 2022: **19**.

125. Pozo-Cano MD, Martín-Salvador A, Pérez-Morente MÁ, Martínez-García E, Castillo JDL, Gázquez-López M, et al. Validation of the women’s views of birth labor satisfaction questionnaire (Womblsq4) in the spanish population. *Int J Environ Res Public Health* 2020: **17**: 1-16.

126. Qian X, Wang Q, Ou X, Li P, Zhao B, Liu H. Effects of Ropivacaine in Patient-Controlled Epidural Analgesia on Uterine Electromyographic Activities during Labor. *BioMed Research International* 2018: 1-7.

127. Rahimi-Kian F, Shahbazi S, Mohammadi S, Haghani S. The effects of ice pack application on pain intensity in the active phase of labor and on birth satisfaction among primiparous women. *Nursing Practice Today* 2018: **5**: 355-62.

128. Ramnerö A, Hanson U, Kihlgren M. Acupuncture treatment during labour -- a randomised controlled trial. *BJOG* 2002: **109**: 637-44.

129. Rao ZA, Choudhri A, Naqvi S, Ehsan Ul H. Walking epidural with low dose bupivacaine plus tramadol on normal labour in primipara. *J Coll Physicians Surg Pak* 2010: **20**: 295-8.

130. Redshaw M, Martin CR. Validation of a perceptions of care adjective checklist. *J Eval Clin Pract* 2009: **15**: 281-8.

131. Redshaw M, Martin CR, Savage-McGlynn E, Harrison S. Women's experiences of maternity care in England: preliminary development of a standard measure. *BMC Pregnancy Childbirth* 2019: **19**: 167.

132. Richardson MG, Lopez BM, Baysinger CL. Should Nitrous Oxide Be Used for Laboring Patients? *Anesthesiol Clin* 2017: **35**: 125-43.

133. Rolinska AA, Tomasz G, Kwasniewska A, Makara-Studzinska M. Searching for Medical Substances Safe for Mother and Child, Facilitating the Delivery of Pain Management and Decreasing Exhaustion--Evaluation of Obstetric Gel by Pregnant Women. *Pain Manag Nurs* 2015: **16**: 493‐8.

134. Romero-Gonzalez B, Peralta-Ramirez MI, Caparros-Gonzalez RA, Cambil-Ledesma A, Hollins Martin CJ, Martin CR. Spanish validation and factor structure of the Birth Satisfaction Scale-Revised (BSS-R). *Midwifery* 2019: **70**: 31-7.

135. Salim R, Nachum Z, Moscovici R, Lavee M, Shalev E. Continuous compared with intermittent epidural infusion on progress of labor and patient satisfaction. *Obstet Gynecol* 2005: **106**: 301-6.

136. Santana LS, Gallo RB, Ferreira CH, Duarte G, Quintana SM, Marcolin AC. Transcutaneous electrical nerve stimulation (TENS) reduces pain and postpones the need for pharmacological analgesia during labour: a randomised trial. *J Physiother* 2016: **62**: 29-34.

137. Sawyer A, Rabe H, Abbott J, Gyte G, Duley L, Ayers S, et al. Measuring parents' experiences and satisfaction with care during very preterm birth: a questionnaire development study. *Bjog-Int J Obstet Gy* 2014: **121**: 1294-301.

138. Shaban I, Mohammad K, Homer C. Development and Validation of Women's Satisfaction With Hospital-Based Intrapartum Care Scale in Jordan. *J Transcult Nurs* 2016: **27**: 256-61.

139. Shapiro A, Fredman B, Zohar E, Olsfanger D, Jedeikin R. Delivery room analgesia: An analysis of maternal satisfaction. *Int J Obstet Anesth* 1998: **7**: 226-30.

140. Shaw-Battista J. Systematic Review of Hydrotherapy Research: Does a Warm Bath in Labor Promote Normal Physiologic Childbirth? *J Perinat Neonatal Nurs* 2017: **31**: 303-16.

141. Shivanagappa M, Kumararadhya GB, Thammaiah SH, Swamy AHM, Suhas N. Progress of labor and obstetric outcome in parturients with combined spinal-epidural analgesia for labor: A comparative study. *Ann Afr Med* 2021: **20**: 270-5.

142. Simavli S, Kaygusuz I, Gumus I, Usluogullari B, Yildirim M, Kafali H. Effect of music therapy during vaginal delivery on postpartum pain relief and mental health. *J Affect Disord* 2014: **156**: 194-9.

143. Simkin PP, O'Hara M. Nonpharmacologic relief of pain during labor: systematic reviews of five methods. *Am J Obstet Gynecol* 2002: **186**: S131-59.

144. Simkin P, Bolding A. Update on nonpharmacologic approaches to relieve labor pain and prevent suffering. 2004: **49**: 489-504.

145. Škodová Z, Nepelová Z, Grendár M, Bašková M. Psychometric properties of the Slovak version of the Birth Satisfaction Scale (BSS) and Birth Satisfaction Scale-Revised (BSS-R). *Midwifery* 2019: **79**: N.PAG-N.PAG.

146. Smith CA, Collins CT, Cyna AM, Crowther CA. Complementary and alternative therapies for pain management in labour. *Cochrane Database Syst Rev* 2006: N.PAG-N.PAG.

147. Smith LA, Burns E, Cuthbert A. Parenteral opioids for maternal pain management in labour. *Cochrane Database Syst Rev* 2018: **6**: Cd007396.

148. Smith CA, Levett KM, Collins CT, Dahlen HG, Ee CC, Suganuma M. Massage, reflexology and other manual methods for pain management in labour. *Cochrane Database Syst Rev* 2018: **2018**.

149. Smith CA, Levett KM, Collins CT, Armour M, Dahlen HG, Suganuma M. Relaxation techniques for pain management in labour. *Cochrane Database Syst Rev* 2018: **3**: Cd009514.

150. Stadlmayr W, Schneider H, Amsler F, Burgin D, Bitzer J. How do obstetric variables influence the dimensions of the birth experience as assessed by Salmon's item list (SIL-Ger)? *Eur J Obstet Gynecol Reprod Biol* 2004: **115**: 43-50.

151. Al Sulmi ES, Al Yousef MM, Almuslim JA, Al Muslim RA, Amailo ZA, Alabbad FA. Awareness, Perceptions, and Desirability of Epidural Analgesia Among Pregnant Women in King Fahad University Hospital, Al Khobar. *Cureus* 2021: **13**: e20146.

152. Taavoni S, Abdolahian S, Haghani H. Effect of sacrum-perineum heat therapy on active phase labor pain and client satisfaction: a randomized, controlled trial study. *Pain Med* 2013: **14**: 1301-6.

153. Taheri M, Takian A, Taghizadeh Z, Jafari N, Sarafraz N. Creating a positive perception of childbirth experience: systematic review and meta-analysis of prenatal and intrapartum interventions. *Reprod Health* 2018: **15**: 73.

154. Thomson G, Feeley C, Moran VH, Downe S, Oladapo OT. Women's experiences of pharmacological and non-pharmacological pain relief methods for labour and childbirth: a qualitative systematic review. *Reproductive Health* 2019: **16**: N.PAG-N.PAG.

155. Thuvarakan K, Zimmermann H, Mikkelsen MK, Gazerani P. Transcutaneous Electrical Nerve Stimulation As A Pain-Relieving Approach in Labor Pain: A Systematic Review and Meta-Analysis of Randomized Controlled Trials. *Neuromodulation* 2020: **23**: 732-46.

156. Tiznobaik A, Geranmayeh M, Khakbazan Z, Taghizadeh Z. Factors affecting maternal satisfaction with vaginal delivery: A qualitative study. *Revista Latinoamericana de Hipertension* 2019: **14**: 277-83.

157. Tournaire M, Theau-Yonneau A. Complementary and alternative approaches to pain relief during labor. *Evid Based Complement Alternat Med* 2007: **4**: 409-17.

158. Tsui MH, Ngan Kee WD, Ng FF, Lau TK. A double blinded randomised placebo-controlled study of intramuscular pethidine for pain relief in the first stage of labour. *BJOG* 2004: **111**: 648-55.

159. Ulfsdottir H, Saltvedt S, Georgsson S. Women's experiences of waterbirth compared with conventional uncomplicated births. *Midwifery* 2019: **79**: N.PAG-N.PAG.

160. Ullman R, Smith LA, Burns E, Mori R, Dowswell T. Parenteral opioids for maternal pain relief in labour. *Cochrane Database Syst Rev* 2010: N.PAG-N.PAG.

161. Unalmis Erdogan S, Yanikkerem E, Goker A. Effects of low back massage on perceived birth pain and satisfaction. *Complement Ther Clin Pract* 2017: **28**: 169-75.

162. van der Spank JT, Cambier DC, De Paepe HM, Danneels LA, Witvrouw EE, Beerens L. Pain relief in labour by transcutaneous electrical nerve stimulation (TENS). *Arch Gynecol Obstet* 2000: **264**: 131-6.

163. van Stenus CMV, Boere-Boonekamp MM, Kerkhof E, Need A. Client experiences with perinatal healthcare for high-risk and low-risk women. *Women Birth* 2018: **31**: e380-e8.

164. Vixner L, Martensson LB, Schytt E. Acupuncture with manual and electrical stimulation for labour pain: a two month follow up of recollection of pain and birth experience. *BMC Complement Altern Med* 2015: **15**: 180.

165. Waldenstrom U, Brown S, McLachlan H, Forster D, Brennecke S. Does team midwife care increase satisfaction with antenatal, intrapartum, and postpartum care? A randomized controlled trial. *Birth* 2000: **27**: 156-67.

166. Walker KF, Wilson P, Bugg GJ, Dencker A, Thornton JG. Childbirth experience questionnaire: validating its use in the United Kingdom. *BMC Pregnancy Childbirth* 2015: **15**: 86.

167. Weng MH, Chou HC, Liaw JJ. Women's sense of control during labour and birth with epidural analgesia: A qualitative descriptive study. *Midwifery* 2023: **116**: 103496.

168. Weibel S, Jelting Y, Afshari A, Pace NL, Eberhart LH, Jokinen J, et al. Patient-controlled analgesia with remifentanil versus alternative parenteral methods for pain management in labour. *Cochrane Database Syst Rev* 2017: **4**: Cd011989.

169. Westergren A, Edin K, Lindkvist M, Christianson M. Exploring the medicalisation of childbirth through women's preferences for and use of pain relief. *Women Birth* 2021: **34**: e118-e27.

170. Wilde-Larsson B, Larsson G, Kvist LJ, Sandin-Bojo AK. Womens' opinions on intrapartal care: development of a theory-based questionnaire. *J Clin Nurs* 2010: **19**: 1748-60.

171. Yeung MPS, Tsang KWK, Yip BHK, Tam WH, Ip WY, Hau FWL, et al. Birth ball for pregnant women in labour research protocol: a multi-centre randomised controlled trial. *BMC Pregnancy Childbirth* 2019: **19**: 153.

172. Xing JJ, Liu XF, Xiong XM, Huang L, Lao CY, Yang M, et al. Effects of Combined Spinal-Epidural Analgesia during Labor on Postpartum Electrophysiological Function of Maternal Pelvic Floor Muscle: A Randomized Controlled Trial. *PLoS One* 2015: **10**: e0137267.

173. Yildirim G, Sahin NH. The effect of breathing and skin stimulation techniques on labour pain perception of Turkish women. *Pain Res Manag* 2004: **9**: 183-7.

174. Yurashevich M, Carvalho B, Butwick AJ, Ando K, Flood PD. Determinants of women's dissatisfaction with anaesthesia care in labour and delivery. *Anaesthesia* 2019: **74**: 1112-20.

175. Zhu X, Wang Y, Zhou H, Qiu L, Pang R. Adaptation of the Childbirth Experience Questionnaire (CEQ) in China: A multisite cross-sectional study. *PLoS One* 2019: **14**: e0215373.
