# Supplementary material for: Pharmacological pain relief and women´s birth experience: a systematic review
Source: BMC Pregnancy Childbirth. 2025 Apr 26;25:505. doi: 10.1186/s12884-025-07602-3 (PMC12032825; doi:10.1186/s12884-025-07602-3)
Supplement: Supplementary file 2 — Supplementary Material 2 [file 12884_2025_7602_MOESM2_ESM.docx]

**Additional file 4**

| **GRADE domain** | **Judgement** | **Concerns about certainty domains** |
| --- | --- | --- |
| Methodological limitations of the studies | One of the two included RCT had a high risk of bias due to inadequate randomisation process, and one had moderate risk of bias due to uncertainty concerning deviations from intended intervention.  Of the 16 observational studies, five had an overall high risk of bias, mainly due to loss to follow up and risk of cofounding. | Serious |
| Indirectess | The outcome was assessed by many different methods in the included studies. The population in the study varied in matter of percentage of vaginal delivery. In several studies the intervention and comparator groups were not clearly separated. | Serious |
| Imprecision | The number of participants in the included studies varied from 70 to 4192. In eight of the 18 included studies the number of participants were <400.  The majority of the included studies were conducted in western high income countries | Serious |
| Inconsistency | The direction and magnitude of effect varied across studies. The smaller observational studies tended to show a negative effect of the intervention on the outcome. | Not serious, borderline |
| Publication bias | The search was comprehensive. However, no grey literature was sought. Both positive and negative results were published |  |

**Certainty of evidence, GRADE approach**

| Outcome | Effect | Certainty of evidence |
| --- | --- | --- |
| Birth satisfaction  Assessed using a variety of scales | Most studies showed a negative effect or no effect of pharmacological pain relief | VERY LOW  XOOO  (due to risk of bias*, indirectness ¤ and imprecision #) |

The outcome of interest was Birth Satisfaction, for which a single pooled effect estimate was not available and only a narrative synthesis of the evidence was provided.

* Serious risk of bias across studies because inadequate randomisation process, inadequate handling of risk of confounding and large loss to follow up.

¤ Serious indirectness as the populations varied in the included studies, and the outcome was not uniformly assessed.

# Serious imprecision were considered due to a large amount of small studies, which was mainly conducted in high-income countries.
