# Supplementary material for: Pharmacological pain relief and women´s birth experience: a systematic review
Source: BMC Pregnancy Childbirth. 2025 Apr 26;25:505. doi: 10.1186/s12884-025-07602-3 (PMC12032825; doi:10.1186/s12884-025-07602-3)
Supplement: Supplementary file 3 — Supplementary Material 3 [file 12884_2025_7602_MOESM3_ESM.docx]

**Additional file 5.** Instruments used in included studies for measuring satisfaction with birth experience.

| **Name of instrument/ acronym/** | **year of publication/**  **author** | **Collection time/no of items** | **Inclusion criteria at time of validation of the tool** | **Used by authors in this review** |
| --- | --- | --- | --- | --- |
| Visual Analogue Scale (VAS) for Birth Satisfaction |  | Mostly used as a Numerical Rating  Scale (NRS) on a 1-10 Likert scale | Postpartum | Larsson |
| Wijma Delivery Expectancy (A)and Experience (B) Questionnaire (W DEQ-B) | 1998  Wijma et al | 24 hrs/  33 items  0-5 Likert scale  Max 165 p | >18 years  No language barrier | Fenaroli  Larsson |
| Childbirth Experience Questionnaire (CEQ) | 2010  Dencker et al | 1 month/  22 items  1-4 Likert- scale | Nulliparous  37-42 weeks  Singleton fetus  Uncomplicated pregnancy  Spontaneous on-set of delivery. | Lathrop |
| Salmon´s Item List (SIL) | 1990  Salmon et al | Postnatal/  20 items  1-7 Likert scale  >70 indicate a positive experience | Primiparous | Spaich |
| Greater expectations | 2006  Baston et al | 3 years/  26 open and 140 closed questions | Participants in the study by Baston were pregnant woman expecting a baby in year 2000 | Rijnders |
| ”Uribe scale” | 2008  Chile  Uribe et al | Postpartum/  42 items  Max 210 p  >172 is defined as “optimal score” | Postpartum women | Weeks |
| Mackey Satisfaction Childbirth Rating Scale | 2004  Mackey and Goodman | Postpartum/ The final score obtained by adding scores from 6 subscales. 34-items in total. Each item presented as dissatisfaction or satisfaction on a 1-5 Likert scale | Low-risk postpartum women, aged 18-46 years, with uneventful vaginal deliveries of healthy full-term infants. | Fernandez- Arranz |
| Birth Satisfaction Scale – Revised (BSS-R) | 2014  Hollins Martin and Martin | Postpartum/  10 items , 3 subscales. 0-4 for each of the 10-items = Top total score of 40. | Postpartum women | Such |
