# Supplementary figures and images for: Pharmacological pain relief and women´s birth experience: a systematic review
Source: BMC Pregnancy Childbirth. 2025 Apr 26;25:505. doi: 10.1186/s12884-025-07602-3 (PMC12032825; doi:10.1186/s12884-025-07602-3)

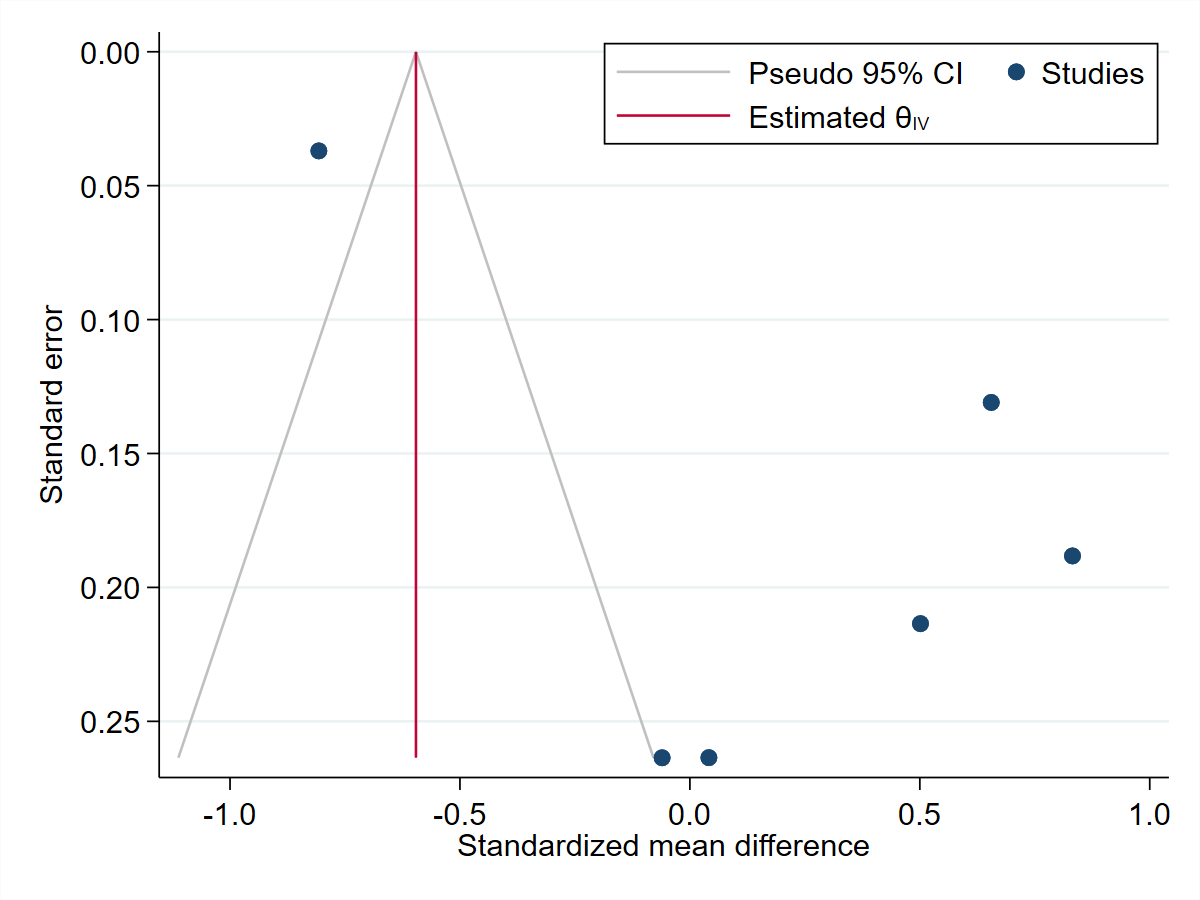

Supplement: Supplementary file 4 — Supplementary Material 4 [file 12884_2025_7602_MOESM4_ESM.png]

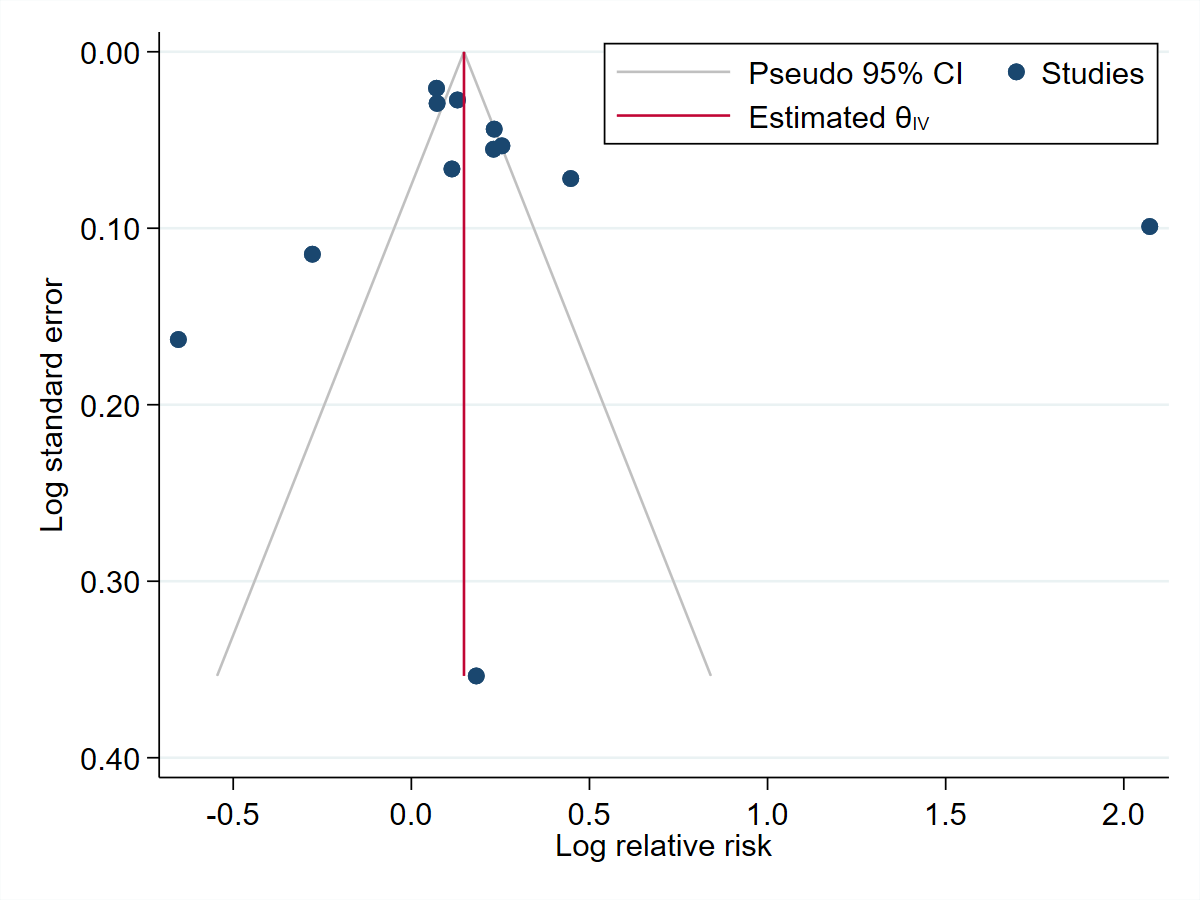

Supplement: Supplementary file 5 — Supplementary Material 5 [file 12884_2025_7602_MOESM5_ESM.png]
