# Supplementary material for: Pharmacological pain relief and women´s birth experience: a systematic review
Source: BMC Pregnancy Childbirth. 2025 Apr 26;25:505. doi: 10.1186/s12884-025-07602-3 (PMC12032825; doi:10.1186/s12884-025-07602-3)
Supplement: Supplementary file 6 — Supplementary Material 6 [file 12884_2025_7602_MOESM6_ESM.docx]

**Summary of number of references in the first search and the three updates.**

| **First search 20190211** | | |
| --- | --- | --- |
| 1 | Total number of results 19980101-20181231 | 3,646 |
| 2 | After duplicate sorting | 2,179 |
| **Update 1. 20191027** | | |
| 3 | Total number of results 20190101-20191027 | 300 |
| 4 | After duplicate sorting | 175 |
| **Update 2. 20200818** | | |
| 5 | Total number of results 20191028-20200819 | 386 |
| 6 | After duplicate sorting | 235 |
| **Update 3. 20230103** | | |
| 7 | Total number of results 20200820-20230103 | 930 |
| 8 | After duplicate sorting | 621 |
| **Combined sets** | | |
| 9 | 1 OR 3 OR 5 OR 7 (total number of results) | 5,262 |
| 10 | 2 OR 4 OR 6 OR 8 (after duplicate sorting) | 3,206 |

**PubMed via NLM**

| #1 | ((((((((("Parturition"[Mesh] OR "Labor, Obstetric"[Mesh] OR "Delivery, Obstetric"[Mesh]) OR ("vaginal delivery"[Title/Abstract] OR "obstetric delivery"[Title/Abstract] OR "bear down"[Title/Abstract] OR labor[Title/Abstract] OR "child birth"[Title/Abstract] OR parturition[Title/Abstract] OR parturient[Title/Abstract] OR birth[Title/Abstract])))))) |
| --- | --- |
| #2 | (((("Pain"[Mesh] OR "Analgesia"[Mesh] OR (pain[Title/Abstract] OR analgesia[Title/Abstract])))) OR (("Labor Pain"[Mesh]) OR "Analgesia, Obstetrical"[Mesh]) OR ("labor pain"[Title/Abstract] OR "obstetrical analgesia"[Title/Abstract]))))))) |
| #3 | ((("Patient Satisfaction"[Mesh]) OR ("patient satisfaction"[Title/Abstract] OR "maternal satisfaction"[Title/Abstract] OR "personal satisfaction"[Title/Abstract] OR "women satisfaction"[Title/Abstract] OR "birth experience"[Title/Abstract])))) |
| #4 | 1 AND 2 AND 3 |
| #5 | english[Filter] AND 1998-[pdat**]** |

**Embase via Elsevier**

| #1 | 'birth'/exp OR 'birth' OR 'childbirth'/exp OR 'childbirth' OR 'labor'/exp OR 'labor' OR 'obstetric delivery'/exp OR 'obstetric delivery' OR 'vaginal delivery':ti,ab OR 'obstetric delivery':ti,ab OR 'bear down':ti,ab OR labor:ti,ab OR 'childbirth':ti,ab OR parturition:ti,ab OR parturient:ti,ab OR birth:ti,ab |
| --- | --- |
| #2 | 'pain'/exp OR 'labor pain'/exp OR 'obstetric analgesia'/exp OR 'analgesia'/exp OR pain:ti,ab OR analgesia:ti,ab OR 'labor pain':ti,ab OR 'obstetrical analgesia':ti,ab |
| #3 | 'patient attitude'/exp OR 'patient satisfaction':ti,ab OR 'birth experience':ti,ab OR 'maternal satisfaction':ti,ab OR 'women satisfaction':ti,ab OR 'personal satisfaction':ti,ab |
| #4 | #1 AND #2 AND #3 |
| #5 | #4 NOT 'conference abstract'/it AND ([english]/lim) |

**Cinahl via Ebsco**

| 1 | MH "Labor+" OR MH "Delivery, Obstetric+" OR MH "Childbirth+" OR (TI “Vaginal delivery” OR “Obstetric delivery” OR “Bear down” OR Labor OR "Childbirth" OR Parturition OR Parturient OR Birth) OR ( AB “Vaginal delivery” OR “Obstetric delivery” OR “Bear down” OR Labor OR "Childbirth" OR Parturition OR Parturient OR Birth) |
| --- | --- |
| 2 | MH "Pain+" OR MH "Labor Pain" OR MH "Analgesia, Obstetrical" OR  MH "Analgesia+" OR (TI Pain OR Analgesia OR “Labor pain” OR “Obstetrical analgesia”) OR (AB Pain OR Analgesia OR “Labor pain” OR “Obstetrical analgesia”) |
| 3 | MH "Patient Satisfaction+" OR (TI “Patient satisfaction” OR “Maternal satisfaction” OR “Personal satisfaction” OR “Women satisfaction” OR “Birth experience”) OR (AB “Patient satisfaction” OR “Maternal satisfaction” OR “Personal satisfaction” OR “Women satisfaction” OR “Birth experience”) |
| 4 | 1 AND 2 AND 3 |
|  | Limiters - Peer Reviewed; English Language; |

**Cochrane via WIley**

| #1 | MeSH descriptor: [Parturition] explode all trees |
| --- | --- |
| #2 | MeSH descriptor: [Labor, Obstetric] explode all trees |
| #3 | MeSH descriptor: [Delivery, Obstetric] explode all trees |
| #4 | ("Vaginal delivery" OR "Obstetric delivery" OR "Bear down" OR Labor OR "Childbirth" OR Parturition OR Parturient OR Birth):ti,ab,kw |
| #5 | {OR #1-#4} |
| #6 | MeSH descriptor: [Pain] explode all trees |
| #7 | MeSH descriptor: [Labor Pain] explode all trees |
| #8 | MeSH descriptor: [Analgesia, Obstetrical] explode all trees |
| #8 | MeSH descriptor: [Analgesia] explode all trees |
| #9 | (Pain OR Analgesia OR "Labor pain" OR "Obstetrical analgesia"):ti,ab,kw |
| #10 | {OR #6-#9} |
| #11 | MeSH descriptor: [Patient Satisfaction] explode all trees |
| #12 | ("Patient satisfaction" OR "Maternal satisfaction" OR "Personal satisfaction" OR "Women satisfaction" OR "Birth experience"):ti,ab,kw |
| #13 | {OR #11-#12} |
| #14 | #5 AND #10 AND #13 |
